# Generative AI and Blockchain-Integrated Multi-Agent Framework for Resilient and Sustainable Fruit Cold-Chain Logistics

**DOI:** 10.3390/foods14173004

**Published:** 2025-08-27

**Authors:** Abhirup Khanna, Sapna Jain, Anushree Sah, Sarishma Dangi, Abhishek Sharma, Sew Sun Tiang, Chin Hong Wong, Wei Hong Lim

**Affiliations:** 1 School of Computer Science, UPES, Dehradun 248007, India; akhanna@ddn.upes.ac.in; 2 Applied Science Cluster (Chemistry), School of Advanced Engineering, UPES, Dehradun 248007, India; sjain@ddn.upes.ac.in; 3 Department of Computer Science and Engineering, Graphic Era Deemed to Be University, Dehradun 248002, India; sarishma.cse@geu.ac.in (S.D.); abhisheksharma.cse@geu.ac.in (A.S.); 4 Faculty of Engineering, Technology and Built Environment, UCSI University, Kuala Lumpur 56000, Malaysia; tiangss@ucsiuniversity.edu.my; 5 Maynooth International Engineering College, Maynooth University, W23 A3HY Maynooth, Ireland; chinhong.wong@mu.ie; 6 Maynooth International Engineering College, Fuzhou University, Fuzhou 350116, China

**Keywords:** cold-chain logistics, multi-agent reinforcement learning, generative AI, blockchain, sustainable food systems

## Abstract

The cold-chain supply of perishable fruits continues to face challenges such as fuel wastage, fragmented stakeholder coordination, and limited real-time adaptability. Traditional solutions, based on static routing and centralized control, fall short in addressing the dynamic, distributed, and secure demands of modern food supply chains. This study presents a novel end-to-end architecture that integrates multi-agent reinforcement learning (MARL), blockchain technology, and generative artificial intelligence. The system features large language model (LLM)-mediated negotiation for inter-enterprise coordination, Pareto-based reward optimization balancing spoilage, energy consumption, delivery time, and climate and emission impact. Smart contracts and Non-Fungible Token (NFT)-based traceability are deployed over a private Ethereum blockchain to ensure compliance, trust, and decentralized governance. Modular agents—trained using centralized training with decentralized execution (CTDE)—handle routing, temperature regulation, spoilage prediction, inventory, and delivery scheduling. Generative AI simulates demand variability and disruption scenarios to strengthen resilient infrastructure. Experiments demonstrate up to 50% reduction in spoilage, 35% energy savings, and 25% lower emissions. The system also cuts travel time by 30% and improves delivery reliability and fruit quality. This work offers a scalable, intelligent, and sustainable supply chain framework, especially suitable for resource-constrained or intermittently connected environments, laying the foundation for future-ready food logistics systems.

## 1. Introduction

The fruit cold chain has been an unavoidable tool in ensuring quality of goods, safety, and freshness at the origin to the final consumption point. It consists of modular temperature-controlled handling and transportation modules developed to slow down spoilage, reduce wastage, and extend the market shelf life, thus providing the produce a proper arrival to its destination. This necessity is especially urgent in the case of export processes where the extended logistic delays can deteriorate the quality of goods without the presence of a healthy cold-chain management. However, the industry faces various challenges. Time fluctuations of the temperature control generated by the failure of equipment, power, or improper handling method enables the degradation of products. Also, few cold storage facilities and refrigerated transportation are hampered by the lack of critical infrastructure particularly in less-developed areas. High energy consumption and related impact on the environment linked to refrigeration increase the urge to employ more sustainable practices. Operation inefficiency can also be brought about by the complexity of this sector, which is the consequence of the need to coordinate different players (producers, logistics providers, and retailers). Therefore, mitigating loss, meeting consumer expectations, and enhancing global food security should become the key priorities when coping with these interimmersive issues [1,2,3,4,5,6,7,8,9,10,11,12,13,14,15,16,17]. The requirement of high quality and fresh fruits in the world market has grown due to the increased health awareness and the growth of the global markets. Bioactive properties of strawberry, bananas, and oranges are also highly expensive, but they are highly perishable and thus sensitive to changes in temperature and humidity. Strawberries require cold storage at temperatures of zero to four degrees Celsius to prevent growth of microorganisms; bananas are vulnerable to ethylene-mediated over-ripening; and oranges are vulnerable to cold injury in an inappropriate environment. To ensure that such high standards of preservation are maintained throughout geographically distributed supply chains adds logistical, economic, and environmental complexities of a considerable nature. The existing state of cold-chain logistics is fraught by such issues as improperly designed routing plans, dysfunctional coordination of stakeholders, high power expenditure, and insufficient end-to-end tracking. The rule-based protocols used in these systems are passive and mainly centralized; therefore, they cannot easily be changed in real time to respond to the changes that may occur to the system such as traffic delays, equipment malfunction, or changes in demand. Additionally, lack of common digital infrastructure contributes to siloed decision-making, eliminates the possibility of accountability, and hampers the chances of optimizing through collaboration. Blockchain technology is fast transforming the supply chain with improved transparency, traceability, and security. The system allows sharing of the information in real time with the involved stakeholders thus a reduction in fraud and inefficiencies. In its turn, blockchain receives an even greater emphasis in the field of finances, healthcare, and law when the technology supports safe transactions, ensures the integrity of data, and provides the implementation of smart contracts to enable automatized activities [18,19,20,21,22,23,24,25,26,27,28,29]. The need to implement decentralized, collaborative solution in complex environments activates the further spread of multiple agent systems. These systems are made of self-governing agents who interact in order to achieve some common or self-centered objectives and in so doing enhance flexibility and scalability. They find more applications in logistics, robotics, or AI-enabled simulations where they have proved to have a higher efficiency, flexibility, and decision-making ability in dynamic fields [30,31,32,33,34,35,36,37,38,39,40,41,42,43,44,45]. The paper suggests a unified AI-powered system of perishable fruit cold-chain logistics based on a composite of modular multi-agent reinforcement learning (MARL), blockchain-driven governance, and generative artificial intelligence. The framework develops three major innovations, namely, (1) LLM-supported negotiation protocols, which will help autonomous agents across different organizations to establish a consensus in terms of creating schedules of deliveries and inventory balancing; (2) the alignment of the Pareto principle to the molding of the reward structures of the RL models so that agents can find some optimal solutions to conflicts between such objectives as the minimization of spoilage, efficient use of energy, adherence to the delivery timelines, and carbon footprint; (3) the application of lightweight agents. In sum, all these factors make up a strong, de-centralized and sustainable logistics system with capabilities to dynamically respond to the variability in the real world. The given architecture is trained on the synthetic but realistic farm-to-retail shipment datasets, and it has included the disruption scenarios made by different transformer-based forecasting models and large language models (LLMs). The system is scalable and can run well in limited-resource or disconnected environments. Finally, it promotes the overall objectives of sustainable, transparent, and efficient food systems by means of intelligent automation and digital trust stacks, as well as context-specific streamlining. The list of acronyms mentioned in this work are presented in Appendix A as Table A1.

### 1.1. Key Contributions

- Multi-Agent Specialization: Designed modular agents for specific tasks such as routing, refrigeration control, spoilage prediction, inventory planning, and delivery scheduling, each using tailored RL algorithms (Q-Learning, DDPG, and GNN-based actor–critic).
- Centralized Training with Decentralized Execution (CTDE): Adopted a CTDE paradigm enabling globally coordinated training and autonomous edge-level decision-making.
- Generative-AI-Enhanced Simulations: Incorporated transformer-based demand forecasting and LLM-generated disruption scenarios to train agents under realistic and extreme edge conditions.
- Blockchain-Enabled Trust Layer: Implemented smart contracts, decentralized identifiers (DIDs), and performance-based incentive systems on a private Ethereum blockchain to enforce SLAs and support transparent collaboration.
- Sustainability Optimization: Introduced a Pareto-based multi-objective reward structure balancing spoilage, delivery timeliness, energy usage, and carbon emissions, allowing context-aware trade-offs.
- Unlike existing MARL-based cold-chain systems, our framework uniquely integrates LLM-mediated negotiations, NFT-based traceability, Pareto-optimized RL, and real-time edge AI within a private blockchain infrastructure, offering a robust and auditable solution for next-generation food logistics.

### 1.2. Paper Organization

The paper is organized as follows. Section 2 provides an account of the methodology. The details of the datasets, the training of the reinforcement learning model, and implementation of a blockchain are described in Section 2.1, Section 2.2 and Section 2.3, correspondingly. In Section 3, the overview of the system architecture, it has been separated with edge, coordination, and enterprise layers. Section 4 expounds on the modular multi-agent reinforcement learning (MARL) framework in terms of agent specialization (Section 4.1), CTDE methodology (Section 4.2), heterogeneous policy design (Section 4.3), and the Pareto-based reward framework (Section 4.4). Section 5 presents the scenario simulation of using generative AI to make agents more adaptable. Section 6 discusses the blockchain as a layer, the smart contracts activities, and the traceability features, such as NFT certification. Section 7 provides performance analyses in terms of different metrics in an experiment. The study is concluded in Section 8, which emphasizes its sustainability in terms of AI-based food systems and the possible future expansion of the study to wider areas of its application and practical implementation.

## 2. Methodology

### 2.1. Data Collection and Synthetic Dataset Generation

It has been difficult to design an environment with lightweight and data-driven reinforcement learning interventions in cold-chain logistics with limited resources only on the storage and computing end. In order to deal with this issue, a synthetic dataset was built that simulates the major dynamics of cold-store management of perishable goods with references to strawberries, bananas, and citrus. Instead of using high-resolution, computationally demanding simulations, the dataset uses simple, memory-sparse models of operational variables so as to reduce redundancy. The parameterization of the dataset was conducted within reasonable bounds and trends using informational inputs, such as agricultural reports by the USDA and FAO, general reports of weather, and case studies of the industry. The shipment episodes recreate the simplified version of the farm-to-retail path and include the records of time-series measures of temperature, humidity, and transit delays, energy use, and simple spoliation indicators. Time-based events like weather conditions and ambient temperature have been coded as discrete categorical properties, or else as a tight numerical range to constrain data size. Values relating to fruit-specific perishability parameters such as strawberries that spoil at temperatures above 40 degree Celsius or bananas that are ethylene sensitive were hardwired as instruction-based flags as opposed to real-time sensor data of a high-frequency [21,29,40,46]. The pre-computed pattern of a supply chain graph along with thresholded risk-scoring based on known degradation profiles were used to create spoilage labels, which need not depend on real-time inference of GNN at the training time. There were also memory savings due to compression of the route and inventory state onto indexed vectors instead of complete event logs and to storage of only notable transition points. The data structure was structured into both a tabular–graph hybrid, thus maintaining a fair representation and execution time. The episodes that collectively make up the shipment amounting to about 10,000 were generated, and each episode has fixed steps (10–20) and features, which makes the dataset few in number to train and test modular RL agents. The controlled noise was injected into demand forecast settled in traffic delays or system failures in order to provide agents with a meaningful set of edge cases and not to overload a system. It is a simpler design that allowed intensive agent training without exceeding the limitations of disk space and the processing capacity. Although smaller, the dataset was able to capture the key variables that affected cold-chain performance, and this enabled the learning of the agents that could be transferred to a new logistics environment. This rationalizes the appropriateness of the dataset in such low-resource experiments and in real-world applications in resource-limited settings in the future.

### 2.2. RL Model Training and Evaluation

In this paper, a reinforcement-learning (RL) system that is specifically built to deal with cold-chain logistics in the perishable-fruits-bearing scenario will be outlined. By use of a modular multi-agent system, every agent will be responsible for one of the specific functions of the system: route planning, temperature control, spoilage estimation, stock control, or delivery scheduling, which leads to task specialization and simplification of the model. Training is carried out using a centralized training and decentralized execution (CTDE) paradigm: agents learn policies using access to a shared global-state during training and are deployed to execute locally observed data. Such real world arrangement has the capability to support scaled, distributed decision-making, a property that is especially beneficial to logistics applications involving intermittent connectivity and localized processes. Since there were also resource limits, lightweight learning algorithms were chosen. Tabular Q-learning is used by the routing agent because of the low overhead cost and discrete action space. A simplicity variant of the Deep Deterministic Policy Gradient (DDPG) algorithm is used as the temperature-control agent and makes the networks smaller and the sampling rate fewer in order to reduce the burden of memory. The spoilage-prediction agent combines a pre-trained Graph Neural Network (GNN) encoder and a shallow actor–critic block to predict the risk of spoilage based upon environmental sensors and fruit-specific perishability curves. Inventory and delivery agents use simplified actor–critic approaches and adopt them to partial-observability and uncertainty in demand. Lastly, agent training is orchestrated by a Pareto-based multi-objective reward function that balances, sometimes contradictory, goals such as spoilage reduction, energy efficiency, carbon footprint and on-time delivery as well as inventory holding costs. The training was carried out on a synthetic dataset with the number of episodes in each task to be 2000 to 5000 (depending on the complexity of a task), and early stopping conditions were used in order to prevent duplicate calculations. The hyperparameters, i.e., learning rate, exploration strategy, and discount factor were optimized using both manual search and task-dependent heuristics. Even with limited computing resources, the RL-trained agents showed significant increases over rule-based and static benchmarks, such as up to 40% reduction in spoilage, more reliable scheduling, improved Service-Level Agreement (SLA) compliance, and more sustainable usage patterns in energy consumption. These results confirm the effectiveness of the RL-based paradigm to improve the cold-chain logistics even when the training resources are scarce, which is why the approach is appropriate to be considered in resource-constrained settings.

### 2.3. Blockchain Implementation Details

A private Ethereum-based blockchain was applied to the proposed cold-chain framework to enhance transparency and ensure data integrity and the ability of decision-making in a decentralized manner between the participating stakeholders. It was chosen due to its well-developed smart-contract application environment, ability to be customized to manage private networks, and suitability with lightweight clients. Understanding the limited storage and computational capacity of the system, the blockchain structure was designed so as to strike a trade-off between trust, automation, and efficiency so that a public chain would not require any overheads. Private Ethereum completely controlled gas fees and block periods, and sources of agreement were rolled out. The structure and architecture allowed the implementation of smart contracts that are resource-efficient and suitable to the cold chain. Important operational data such as an average temperature of shipments, when deliveries were made, and spoilage warnings, as well as an outcome of service-level agreements (SLAs), are captured on-chain as efficient summaries. Off-chain data and high-rate sensor data (e.g., temperature or humidity streams) are stored on-chain, with secure cryptographic hashes of that data stored on-chain to maintain verifiability and limit storage overhead. The high frequency in which environmental sensors measure data, e.g., temperature and humidity, is extremely challenging to compute and store in a blockchain-based system. A hybrid architecture focusing on minimization of transactions has been adopted in order to alleviate these limitations. The readings by the sensors are stored off-chain, either in secured cloud or local databases, whereas their hash digest of cryptography is reflected on-chain. This setup also allows the verifiability and integrity of the initial data, and it prevents prohibitive gas and storage costs of recording all data in full on-chain logging. The smart contracts that will be used in this framework will be created to be as simple as possible, and they should work on the principle of an event-like alert occurrence followed by an action linked to an event-like logging of the performance summary; these smart contracts are the main ones that will enforce SLAs, log performance skeptons, and send out alerts when SLAs are broken. The system attains rapid execution, less gas consumption, and increased throughput in nodes of a private Ethereum network by constraining the complexity of contracts. Solidity included smart contracts in it with very little computation logic in them to improve speed and gas consumption. Examples of modules are (1) a contract to measure the shipment delivery time and condition and compare pre-agreed thresholds leading to fines or rewards; (2) a contract to capture alerts when perishable conditions go above critical limits; and (3) a contract that estimates carbon footprint using total energy usage. These are event-driven and can be called through RESTful APIs, which allow agents to interact with the blockchain asynchronously and without carrying the full Ethereum nodes. Decentralized identifiers (DIDs) were employed to register the participants to enable stakeholder authentication and access control as well as to provide tamper-resistant audit trails. The outcome of the negotiation by the agents or a mutual decision is also noted to bring about co-responsibility in the enterprises. All in all, the Ethereum-based implementation offers secure and traceable digital infrastructure that can be used to automate, monitor, and coordinate stakeholders compatible with low-resource settings. The lightweight, decentralized, and modular design guarantees that the blockchain layer can be hidden in the general cold-chain system without making much calculation and storage expenses.

## 3. System Architecture Overview

The multi-layer approach introduced in the research paper is a multi-level, modular approach to enhancing the resiliency and sustainability of fruit cold-chain logistics with the coherent implementation of multi-agent reinforcement learning (MARL), generative AI, blockchain, and edge computing. The introduced architecture is based on three layers that are planned to be used as the core: edge, coordination, and enterprise with their functions and intra-layer interactions, allowing the realization of global system optimality and implementing real-time decentralized decision-making. At the edge layer, lightweight RL agents are deployed autonomously onto Internet-of-Things (IoT)-enabled infrastructure like smart crates, sensors that are inside vehicles. They are mostly involved in local activities such as mediating temperature, risk of spoilage calculation, and route adjustments in real time. The results are quick responsiveness to environmental changes because the edge layer provides a prompt reaction to real-time sensor information, hence reducing delays and spoilage capacity without the collaboration of a centralized communication system. The coordination level works as an intermediate between the operations (which are heterogeneous at the edge level) and the logistics strategy as a whole. In this layer, service-level agreements (SLAs) are overseen with the help of smart contracts, which are hosted on a dedicated blockchain, together with delivery results and enforcement regulations. The layer also includes large language models (LLMs) that enable the negotiation of agents and integrates modules of generative AI that can simulate change in demand, any disruptions in the supply chain, and any extreme weather conditions. Such simulations brief the agent in training as well as operation and help these agents be more proactive in responding. The enterprise store provides centralized training installations, using the centralized training and decentralized execution (CTDE) model. Based on this position, the agents have the global perspective of the cold-chain system, thus coordinating and aligning the similar objective of reducing waste, optimizing energy use and on-time delivery. Analytics of sustainability, life-cycle assessment, and optimization on multiple dimensions of operational objectives are also obtainable through the enterprise layer. Aggregates of data collected by the edge and coordination levels are fed up to this level, providing iteration and evolution of agent policies. It is the combination of those design features that provide an increased level of adaptability, resilience, and trust on the distributed and heterogeneous supply chain. The framework also centralizes a range of technology and stake holders as well as maintaining secure and transparent processes. This is a type of arrangement that enables an effective cold chain, even in resource-limited or unreliably connected situations, and combines the immediate responsiveness with long-run optimization. Under the described framework, the deliberate transport of information through each layer enables the coherent integration of the locally taken decisions with the general supply-chain smartness. The position of the edge layer is placed in collecting sensor inputs, which include notifications and condition data, e.g., temperature and humidity of control instruments connected to the Internet-of-Things. Edge agents operate on lightweight client implementations that communicate to blockchains through JSON-RPC protocol based interfaces or through Infura-like gateways, running in a private mode that facilitates entirely asynchronousblockchain communications, with no need to deploy full nodes. This architecture supports resource-restrained devices and situations when the connectivity is intermittent. According to initial simulated experimentation, and steady-state tests, every client is assured to process a maximum of 10 transactions per second, and the occurrence of a latency that triggers an SLA is less than 2 s. The lightweight agents at the edges process such readings instantly in determining them, and they are selectively sent off to the coordination layer. Relevant data points in the coordination layer such as the condition of a shipment, warnings about spoilage, and compliance with SLA are noted on a private blockchain through the use of smart contracts. The high-volume stream sensor out-of-chain data are available, but on-chain, the cryptographic hash representations of the out-of-chain streams described represent on-chain traceability and verifiability. In this layer, the coordinating agents also spread summaries and participate in negotiable processes, whose text-based business is seen and brokered by large language models. Aggregated data streams are at the enterprise layer examined in order to tune policy training, to plan the simulation of future scenarios, and to update system-wide goals. A result like demand forecast, sustainability, and disruption scenario analysis is then disseminated to the coordination and the edge layer. These values apply in both the training programs of an agent and the decision-making algorithm of the real-time computing logic. Moreover, with the help of asynchronous and bandwidth non-intensive protocols, the architecture will allow edge agents to work in an autonomous fashion but at the same time maintain operational consistency when placed within operating coherence in the larger supply-chain environment, thus rendering coordination central to the operation.

## 4. Modular Multi-Agent RL Framework

### 4.1. Agent Specializations and Objectives

With agent consensus and cross-company coordination, the present paper proposes a framework that will allow independent, collaborative processes in the cold chain, in particular, organization boundaries. The offered mechanism is crucial to fragmented supply chains where no infrastructure is shared by its actors who are growers, transporters, warehouse operators, and retailers and have no comprehensive visibility. The negotiation point is an LLM-mediated negotiation: the agents of both stakeholders negotiate about delivery times, re-stocks, and change of routes. Offers that are proposed are compared with local utility functions and personal preferences. Through dialogue, the LLM examines past negotiations in order to determine intent, determine areas of common concern, and provide win–win solutions. The summary of the LLM acts as a common ground, which enables the agents to come to an agreement. In case both parties consider the given solution useful, a contract is formulated and signed (using smart contracts on the implementation of a private blockchain). The contracts outline performance measures and compliance requirements, such as acceptable limits of spoilage, delivery time, and quota of carbon emissions. Once a shipment or an assignment regarding an assignment is over, the sensor information and delivery records are written up and contrasted with the consensual conditions. Deposits are made in order to receive rewards depending on the requirements; thus, violations lead to sanctions, improving the criteria of enforcement and openness. Under this system, therefore, operations are automated and risks of dispute minimized. By maintaining autonomy of the agents and promoting collaborative actors, the framework allows both asynchronous communication and decentralized decision-making, which are both desirable traits when a permanently connected environment is not certain. Decentralized identifiers (DIDs) are used to identify agents and allow tracking of their activities on the network safely. This architecture also allows flexible and dynamic reallocation of resources under disruptions: in cases where one warehouse is under increased demand, it can engage other nearby warehouses to balance the inventory. Taken together, those characteristics enable adaptive, peer-to-peer negotiations that do not require a central authority to support supply chain agility and resilience. To conclude, negotiation with LLM mediation, blockchain-enforced agreements, and decentralized identities of agents provides scalable trust-based solution to maintain the complex logistics between independent businesses in the cold chain. Table 1 comprehensively presents an overview of the functional modules, listing inputs, outputs, and reinforcement learning models in the framework. Table 2 details agent roles, observations, and coordination mechanisms, describing multi-agent interactions and distributed decision-making in the cold-chain system.

Algorithm 1 designates a dynamic path selection perishability aware Q-learning routing protocol. It also optimizes the selection of the journeys, which in turn facilitates strong and effective temperature-regulated transportation by calculating rewards that penalize travel time, chance of spoilage, and emissions. Algorithm 2 is a Deep Deterministic Policy Gradient (DDPG) system performed in real-time, crate-level temperature regulation. This model will save energy and reduce spoilage, which will be caused by the thermal deviation, and thus will enable it to attain both energy and product integrity. Algorithm 3 is a junction of Graph Neural Network (GNN) encoding and actor–critic training, and it makes it possible to predict the probabilities of spoilage at a distribution node. All these forecasts make it possible to carry out proactive measures aimed at ensuring that spoilages do not occur as often. Algorithm 4 can implement an actor–critic structure of controlling inventory, with sustainability-driven rewards that ensure trade-offs between carbon footprint, holding costs, and risk of spoilage. Cooperative multi-agent reinforcement learning is used in Algorithm 5 to schedule the delivery and fulfill the service-level agreements, to limit delays, and to minimize emissions by coordinating these actions with joint-policy optimization.
**Algorithm 1** Perishable-Aware Route Optimization via Q-Learning with Context-Aware Weights and Conflict Avoidance  1:**Input:** Cold-chain graph G(V,E), perishability profile *P*, disruption model *D*, emission matrix *C*  2:**Initialize:** Q-table Q(s, a)←0; learning rate α; discount factor γ; exploration rate ϵ  3:**Initialize:** Static priority coefficients $\alpha_{1},\;\alpha_{2},\;\alpha_{3}$  4:**Initialize:** Shared intention buffer B←∅▹ For coordination  5:**for** each episode **do**  6:       Initialize joint global state $S_{0}=\left\{s_{0}^{\left(1\right)},\ldots ,s_{0}^{\left(n\right)}\right\}$ using *D*  7:       **while** shipment not delivered **do**  8:             **for** each routing agent *i* **do**  9:                With probability ϵ, choose random action $a^{\left(i\right)}$10:              Otherwise, choose $a^{\left(i\right)}\leftarrow \arg\max_{a^{\prime }}Q\left(s^{\left(i\right)},\;a^{\prime }\right)$11:                Append $\left(s^{\left(i\right)},\;a^{\left(i\right)}\right)$ to B▹ Declare action intention12:             **end for**13:             Detect conflicts in B (e.g., duplicate vehicle or route allocation)14:             **if** conflict detected **then**15:             Apply coordination penalty ρ or reassign conflicting agent(s) via tie-breaking16:             **end if**17:             **for** each agent *i* **do**18:             Execute $a^{\left(i\right)}$, observe $s^{\prime (i)}$, travel time $t^{\left(i\right)}$, temp deviation $\Delta T^{\left(i\right)}$, emissions $e^{\left(i\right)}$19:             Compute spoilage risk: $\sigma^{\left(i\right)}\leftarrow f\left(P,\;\Delta T^{\left(i\right)}\right)$20:             Extract context vector: $ctx^{\left(i\right)}=\left[\Delta T^{\left(i\right)},\;\mathrm{traffic},\;\mathrm{SLA}\;\mathrm{priority}\right]$21:             Compute dynamic weights:$$\omega_{j}^{\left(i\right)}=\frac{\alpha_{j}\cdot ctx_{j}^{\left(i\right)}}{\sum_{k=1}^{3}\alpha_{k}\cdot ctx_{k}^{\left(i\right)}},\;j=1,2,3$$22:             Compute context-aware reward:$$r^{\left(i\right)}=-\left(\omega_{1}^{\left(i\right)}t^{\left(i\right)}+\omega_{2}^{\left(i\right)}\sigma^{\left(i\right)}+\omega_{3}^{\left(i\right)}e^{\left(i\right)}\right)-\rho$$23:             Update Q-table:$$Q\left(s^{\left(i\right)},\;a^{\left(i\right)}\right)\leftarrow Q\left(s^{\left(i\right)},a^{\left(i\right)}\right)+\alpha \left[r^{\left(i\right)}+\gamma \max_{a^{\prime }}Q\left(s^{\prime (i)},a^{\prime }\right)-Q\left(s^{\left(i\right)},a^{\left(i\right)}\right)\right]$$24:             Update state: $s^{\left(i\right)}\leftarrow s^{\prime (i)}$25:             **end for**26:             Clear intention buffer: B←∅27:    **end while**28:**end for**29:**Output:** Learned policies $\pi_{i}^{*}\left(s\right)=\arg\max_{a}Q\left(s,\;a\right)$ for all agents *i*

**Algorithm 2** Edge-Aware Temperature Control via DDPG with Context-Aware Weights and Coordination
  1:**Input:** Environment state $s=[T_{\mathrm{crate}},\;T_{\mathrm{ambient}},H,\mathrm{fruit}\;\mathrm{type}]$, spoilage model *S*, energy profile *E*  2:**Initialize:** Actor network $\mu (s|\theta^{\mu })$, critic network $Q(s,a|\theta^{Q})$  3:**Initialize:** Target networks $\mu^{\prime }$ and $Q^{\prime }$ with $\theta^{\mu^{\prime }}\leftarrow \theta^{\mu }$, $\theta^{Q^{\prime }}\leftarrow \theta^{Q}$  4:**Initialize:** Replay buffer R, noise process N  5:**Initialize:** Shared intention buffer B←∅, static coefficients $\alpha_{1},\alpha_{2}$  6:**for** each episode **do**  7:   Receive initial global state $S_{0}=\left\{s_{0}^{\left(1\right)},s_{0}^{\left(2\right)},\ldots \right\}$  8:   **for** each timestep *t* **do**  9:          **for** each agent *i* **do**10:             Select action $a_{t}^{\left(i\right)}=\mu \left(s_{t}^{\left(i\right)}|\theta^{\mu }\right)+N_{t}$11:             Append $\left(s_{t}^{\left(i\right)},a_{t}^{\left(i\right)}\right)$ to B12:          **end for**13:          Detect overlapping cooling requests in B (e.g., shared compressor or zone contention)14:          **if** conflict detected **then**15:             Apply coordination penalty ρ or reschedule conflicting setpoints16:          **end if**17:          **for** each agent *i* **do**18:             Apply action $a_{t}^{\left(i\right)}$, observe next state $s_{t+1}^{\left(i\right)}$, energy cost $E_{t}^{\left(i\right)}$, deviation $\Delta T^{\left(i\right)}$, duration Δt19:             Estimate spoilage risk $\sigma_{t}^{\left(i\right)}=S\left(s_{t}^{\left(i\right)},a_{t}^{\left(i\right)},\Delta t\right)$20:             Define context vector: $ctx^{\left(i\right)}=\left[\Delta T^{\left(i\right)},E_{t}^{\left(i\right)},\sigma_{t}^{\left(i\right)}\right]$21:             Compute dynamic weights:$$\omega_{j}^{\left(i\right)}=\frac{\alpha_{j}\cdot ctx_{j}^{\left(i\right)}}{\sum_{k=1}^{3}\alpha_{k}\cdot ctx_{k}^{\left(i\right)}},\;\;j=1,\;2,\;3$$22:             Compute reward:$$r_{t}^{\left(i\right)}=-\left(\omega_{1}^{\left(i\right)}E_{t}^{\left(i\right)}+\omega_{2}^{\left(i\right)}\sigma_{t}^{\left(i\right)}\right)-\rho$$23:             Store transition $\left(s_{t}^{\left(i\right)},a_{t}^{\left(i\right)},r_{t}^{\left(i\right)},s_{t+1}^{\left(i\right)}\right)$ in R24:          **end for**25:          Sample mini-batch from R26:          Update Critic using Bellman loss:$$L={\left(r+\gamma Q^{\prime }\left(s^{\prime },\mu^{\prime }\left(s^{\prime }\right)\right)-Q\left(s,a\right)\right)}^{2}$$27:          Update actor via policy gradient:$$\nabla_{\theta^{\mu }}J\approx \frac{1}{N}\sum \nabla_{a}Q\left(s,a\right)\nabla_{\theta^{\mu }}\mu \left(s\right)$$28:          Soft update target networks:$$\theta^{Q^{\prime }}\leftarrow \tau \theta^{Q}+\left(1-\tau \right)\theta^{Q^{\prime }},\;\;\theta^{\mu^{\prime }}\leftarrow \tau \theta^{\mu }+\left(1-\tau \right)\theta^{\mu^{\prime }}$$29:          Clear B←∅30:      **end for**31:
**end for**
32:**Output:** Trained temperature control policy $\mu^{*}\left(s\right)$


**Algorithm 3** Spoilage Prediction Using GNN and Actor–Critic Learning with Context-Aware Weights and Coordination
  1:**Input:** Cold-chain graph G=(V,E), node features $X_{v}=\left[T,H,\mathrm{delay},\mathrm{fruit}\;\mathrm{type}\right]$, disruption scenarios *D*, spoilage labels *y*  2:**Initialize:** Actor $\mu_{\theta }$, critic $Q_{\phi }$, GNN encoder $f_{\mathrm{GNN}}$, replay buffer R  3:**Initialize:** Shared intention buffer B←∅; static reward coefficients $\alpha_{1},\alpha_{2}$  4:**for** each training episode **do**  5:    Simulate disrupted scenario from *D*  6:    Build graph *G* with node features *X*  7:    Compute graph embedding $z=f_{\mathrm{GNN}}\left(G,X\right)$  8:    Set initial state $s_{0}=z$  9:    **for** each timestep *t* **do**

10:          Select action $a_{t}=\mu_{\theta }\left(s_{t}\right)$
▹ e.g., spoilage threshold or inspection trigger
11:          Append $\left(s_{t},a_{t}\right)$ to B
▹ Declare spoilage alert intention
12:          **if** conflicting inspections or redundant predictions detected in B **then**13:             Apply penalty ρ or adjust decision based on criticality14:          **end if**15:          Predict spoilage: ${\hat{y}}_{t}=\mathrm{sigmoid}\left(Wa_{t}+b\right)$16:          Observe ground truth $y_{t}$ and compute classification loss $L_{\mathrm{pred}}$17:          Compute false negative (FN) rate as critical misprediction signal18:          Extract context vector: $ctx=[L_{\mathrm{pred}},\mathrm{FN},\mathrm{inspection}\;\cos t]$19:          Compute dynamic weights:$$\omega_{j}=\frac{\alpha_{j}\cdot ctx_{j}}{\sum_{k}\alpha_{k}\cdot ctx_{k}},\;\;j=1,2,3$$20:          Compute reward:$$r_{t}=-\left(\omega_{1}\cdot L_{\mathrm{pred}}+\omega_{2}\cdot \mathrm{FN}+\omega_{3}\cdot \cos t\right)-\rho$$21:          Observe next state $s_{t+1}$ from updated GNN encoding22:          Store $\left(s_{t},a_{t},r_{t},s_{t+1}\right)$ in R23:          Sample mini-batch from R24:          Update critic:$$L_{\mathrm{critic}}={\left(r+\gamma Q_{\phi }\left(s^{\prime },\mu_{\theta }\left(s^{\prime }\right)\right)-Q_{\phi }\left(s,a\right)\right)}^{2}$$25:          Update actor:$$\nabla_{\theta }J\approx \nabla_{a}Q_{\phi }\left(s,a\right)\cdot \nabla_{\theta }\mu_{\theta }\left(s\right)$$26:          Optionally fine-tune $f_{\mathrm{GNN}}$ with prediction loss $L_{\mathrm{pred}}$27:          Clear B←∅28:       **end for**29:
**end for**
30:**Output:** Trained spoilage policy $\mu^{*}\left(s\right)$ and GNN encoder $f_{\mathrm{GNN}}^{*}$


### 4.2. Centralized Training with Decentralized Execution (CTDE)

Since the advent of multi-agent reinforcement learning (MARL), the policy of centralized training with decentralized execution (CTDE) has been a fundamental paradigm that allows training cooperatively in a centralized training environment but decoupled execution in real-world distributed environments. Within cold-chain logistics of perishable fruits, the architecture in this paper offers a scalable and insensible strategy of harmonizing the multifarious and asynchronous decisions of routing, inventory, temperature control, spoilage forecasting, and scheduling of deliveries. In the centralized training regime, all agents, i.e., warehouse manager, refrigeration controller, and transport scheduler, have joint access to a global state observing environmental factors including the type of fruits, the prevailing temperature and humidity, delivery conditions, and the impending possibility of spoilage. Such an integrated view allows agents to formulate coordinated policy covering such dependence across the cold chain, thus enabling anticipation and reaction to other agents actions. In complex multi-agent environments, a common critic network could be added in order to secondarily assess collaborative efforts, thus stabilizing training and improving the convergence. Trained separately, these agents will be then implemented in the decentralized way and can be deployed as a cloud-based system or on the edge devices themselves (like on smart crates or sensors in trucks). Agents have local observations, they act in an autonomous manner in this configuration, and they do not require having constant global synchronization or a centralized communication channel. This autonomy is fundamental to cold-chain logistics, the sphere where the connection can be occasionally interrupted, real-time reaction is desirable, and local judgment is limited. The framework of CTDE is very beneficial in the inference of dynamic modules of generative demand simulations, disruption forecasting, and blockchain-based traceability. As another example, a delivery scheduling agent could train on the global awareness of exogenous demand spikes or weather interference simulated by the generative AI model but locally act due to the considerations of real-time traffic and service-level agreement (SLA) constraints. Likewise, an agent that is maximizing global sustainability might consider local emissions of carbon or trash in packaging to make local decisions. In general, CTDE provides increased adaptability, robustness, and scalability in the system and maintains coherence in decisions between choices even when agents have to work beneath uncertain, delayed, or incomplete information. In turn, the paradigm is a suitable option of robust resilient cold-chain implementations with heterogeneous agents and a decentralized infrastructure.
**Algorithm 4** Sustainability-Aware Inventory Management via Actor–Critic RL with Context-Aware Weights and Coordination  1:**Input:** Local inventory state s=[stocklevel,demandforecast,shelflife,carbonscore]  2:**Initialize:** Actor network $\mu_{\theta }\left(s\right)$, critic network $Q_{\phi }\left(s,a\right)$, replay buffer R  3:**Initialize:** Shared intention buffer B←∅, coefficients $\alpha_{1},\alpha_{2},\alpha_{3}$  4:**for** each episode **do**  5:   Observe global inventory state $S=\{s^{\left(1\right)},s^{\left(2\right)},\ldots ,s^{\left(n\right)}\}$ and local state $s_{0}$  6:   **for** each timestep *t* **do**  7:         Select order quantity $a_{t}=\mu_{\theta }\left(s_{t}\right)$  8:         Append $\left(s_{t},a_{t}\right)$ to shared buffer B  9:         **if** conflict detected in B (e.g., stock over-allocation or supply contention) **then**10:             Apply coordination penalty ρ or reassign $a_{t}$11:         **end if**12:         Optionally exchange supply info with peers (e.g., via blockchain or DIDs)13:         Execute $a_{t}$, observe new state $s_{t+1}$14:         Compute spoilage loss $L_{\mathrm{spoil}}$ from overstocked perishables15:         Compute holding cost $H_{t}$ and emissions $E_{t}$ from delivery16:         Define context vector: $ctx=[L_{\mathrm{spoil}},H_{t},E_{t}]$17:         Compute dynamic weights:$$\omega_{j}=\frac{\alpha_{j}\cdot ctx_{j}}{\sum_{k}\alpha_{k}\cdot ctx_{k}},\;j=1,2,3$$18:         Compute reward:$$r_{t}=-\left(\omega_{1}\cdot L_{\mathrm{spoil}}+\omega_{2}\cdot H_{t}+\omega_{3}\cdot E_{t}\right)-\rho$$19:         Store transition $\left(s_{t},a_{t},r_{t},s_{t+1}\right)$ in R20:         Sample mini-batch from R21:         Update critic:$$L_{\mathrm{critic}}={\left(r+\gamma Q_{\phi }\left(s^{\prime },\mu_{\theta }\left(s^{\prime }\right)\right)-Q_{\phi }\left(s,a\right)\right)}^{2}$$22:         Update actor via policy gradient:$$\nabla_{\theta }J\approx E_{s∼R}\left[\nabla_{a}Q_{\phi }\left(s,a\right)\cdot \nabla_{\theta }\mu_{\theta }\left(s\right)\right]$$23:         Clear B←∅24:      **end for**25:**end for**26:**Output:** Trained inventory ordering policy $\mu^{*}\left(s\right)$

**Algorithm 5** SLA-Aware Delivery Scheduling via Cooperative Multi-Agent RL with Context-Aware Weights and Coordination
  1:**Input:** Delivery queue *Q*, route availability *R*, SLA terms *S*, demand forecast *F*  2:**Agents: **$A_{1},A_{2},\ldots ,A_{n}$ (e.g., vehicle or hub controllers)  3:**Initialize:** Policy $\pi_{i}\left(s_{i}\right)$ for each agent *i*, shared critic $Q(s_{1},\ldots ,s_{n},a_{1},\ldots ,a_{n})$  4:**Initialize:** Replay buffer R, intention buffer B←∅  5:**Initialize:** Reward weighting coefficients $\alpha_{1},\;\alpha_{2},\;\alpha_{3}$  6:**for** each training episode **do**  7:   Generate demand and disruptions from *F*  8:   Initialize global state $S_{0}=\left\{s_{0}^{\left(1\right)},\ldots ,s_{0}^{\left(n\right)}\right\}$ from environment  9:   **for** each timestep *t* **do**10:          **for** each agent *i* **do**

11:             Select action $a_{i}=\pi_{i}\left(s_{i}\right)$
▹ e.g., assign vehicle or reschedule
12:             Append $\left(s_{i},a_{i}\right)$ to B13:          **end for**14:          **if** conflicting vehicle assignments or resource overuse in B **then**15:             Apply penalty ρ or resolve using SLA priority or distance heuristics16:          **end if**17:          Execute actions $a=[a_{1},\ldots ,a_{n}]$, observe $s^{\prime }=\left[s_{1}^{\prime },\ldots ,s_{n}^{\prime }\right]$18:          **for** each agent *i* **do**19:             Observe: delay $\delta_{i}$, SLA violation flag $v_{i}$, fuel used $f_{i}$, emissions $e_{i}$20:             Extract context vector: $ctx^{\left(i\right)}=\left[\delta_{i},v_{i},e_{i}\right]$21:             Compute dynamic weights:$$\omega_{j}^{\left(i\right)}=\frac{\alpha_{j}\cdot ctx_{j}^{\left(i\right)}}{\sum_{k}\alpha_{k}\cdot ctx_{k}^{\left(i\right)}},\;j=1,\;2\;,3$$22:             Compute reward:$$r_{i}=-\left(\omega_{1}^{\left(i\right)}\cdot \delta_{i}+\omega_{2}^{\left(i\right)}\cdot v_{i}+\omega_{3}^{\left(i\right)}\cdot e_{i}\right)-\rho$$23:             Store transition $\left(s_{i},a_{i},r_{i},s_{i}^{\prime }\right)$ in R24:          **end for**25:          Sample mini-batch from R26:          Update shared critic *Q* by minimizing temporal-difference loss:$$L={\left(r+\gamma Q\left(s_{1}^{\prime },\ldots ,\;s_{n}^{\prime },\;\pi_{1}\left(s_{1}^{\prime }\right),\ldots ,\;\pi_{n}\left(s_{n}^{\prime }\right)\right)-Q\left(s_{1},\ldots ,\;s_{n},\;a_{1},\ldots ,\;a_{n}\right)\right)}^{2}$$27:          **for** each agent *i* **do**28:             Update actor policy $\pi_{i}$ to maximize expected reward:$$\nabla_{\theta_{i}}J\approx E\left[\nabla_{a_{i}}Q\left(s,a\right)\cdot \nabla_{\theta_{i}}\pi_{i}\left(s_{i}\right)\right]$$29:          **end for**30:          Clear B←∅31:     **end for**32:
**end for**
33:**Output:** Trained delivery policies $\pi_{1}^{*},\ldots ,\pi_{n}^{*}$


### 4.3. Hybrid Heterogeneous Policy Design

Agents in such systems have different, heterogeneous, roles, which include routing, refrigeration control, spoilage prediction, and inventory planning in complex, multi-agent systems like perishable food cold chains. In order to support this diversity, we offer a hybrid heterogeneous design policy, whereby each agent adopts a domain-specific learning structure that corresponds to its functional purpose, horizons in decision-making, act space, and frame obstacles. Unlike the historical homogeneous agent systems where the policy structures are assumed to be the same in all the entities, our system adjusts individual entity policy structures to reflect individual operating properties of the entity. As an example, the route optimization agent has a discrete action space and is learning using Q-learning that has to select an optimal next hop in a transportation network. Comparatively, the temperature control agent chooses a continuous action space by manipulating setpoints of refrigeration through a policy using DDPG. By using graph-structured data that can be used, containing the supply chain checkpoints, the spoilage prediction agent combines a graph-neural-network encoder with an actor–critic reinforcement learning system to adjust the risk thresholds in real time. Agents of inventory management operate within the asymmetric information conditions and employ actor–critic models to decide on the order during the demand uncertainty, local perishability, and sustainability limit, which is possible due to partial observability at warehouse nodes, distribution centers, and retailer shelves. The heterogeneous design in addition to the relationship between the complexity of the model to the structure of the tasks makes it realistic and deployable. Since training each agent requires a different observation and action specification, the framework is modular with the possibility of adding more types of agents in future deployment, e.g., recycling optimizers or demand-side controllers. The different agents are coordinated so that they have different architectures and have decentralized execution that has centralized training so that the agents can learn to behave in common goals and act independent of each other. Single reward functions such as Pareto-weighted penalties calculated based on amount of spoilage, time of delivery, and amount of carbon emission ensure coordination among the agents. On the whole, the hybrid heterogeneous policy design renders flexibility and the optimality of a specific task and generalization even in real-world, dynamic environments of cold chains.

### 4.4. Pareto-Based Multi-Objective Reward Structuring

The nature of real-life cold-chain logistics requires the multiple goals, which are involved with each other, to be pursued simultaneously, i.e., minimum spoilage, minimization of the delay in delivery, energy conservation, service-level agreement, and carbon emission reduction. These trade-offs are easier to overcome in more conventional single-scalar formulations of reward at the cost of possibly suboptimal behavior by the agent under study. The following study overcomes this shortcoming by using a Pareto-based multi-objective structure of rewards according to which each agent will reward actions according to a set of rewards, which represent a measurement of unique performance variables. Agents are taught to distinguish Pareto-optimal actions that solutions to which cannot improve one objective without worsening another; rather than pre-determining a weighting across these goals, agents learn to associate policies with Pareto-optimal actions. An example would be where a delivery agent would conclude that the minor delay they accept will minimize wastes and conserve fuel, hence improving its productivity. Centralized training unites them by feed-backing the resulting vector-based rewards to a common critic network, which can learn in a coordinated fashion. The system is also capable of dynamic weighting of objectives based on factors at hand, given contextual signals like forecast of disruption, level of priority of the customer, or sustainability demands. Reward shaping is used in practice to impose constraints, such as a limit to the temperatures that the system can deviate above or below and service-level agreements with fines that can be associated with smart contracts on blockchain chains to automate constraint enforcement. This architecture allows the agents to change their policies on a concurrent basis and, at the same time, be in line with the general supply-chain goals. When temperatures are high, as in the case at the moment, the reward vectors covering aspects of spoilage might be stressed more, but when the regulatory agencies are cracking the whip on emissions, the sustainability aspects might take center stage. Pareto-based formulation, in turn, promotes adaptability, fairness, and resilience based on endowing all of the agents with the capacity to locally optimize, and contribute to, globally consistent and morally sound decision-making through the cold chain. Such a reward scheme brings about less imbalanced, less geared to misperception policy learning that will ultimately enhance generalization, compliance, and performance in chaotic and uncertain logistics situations.

## 5. Generative AI-Enhanced Scenario Simulation

The contemporary cold-chain logistics requires active, robust decision-making in the routing, inventory control, temperature maintenance, and delivery scheduling. This has to be done through the simulation of realistic operational scenarios. In this respect, the current research proposes a generative artificial-intelligence-assisted simulation component that is going to provide context-sensitive projections concerning demand inconsistency and supply-chain vocations and can be combined with reinforcement-learning processes aimed at training agents. The heart of the framework includes a transformer-based module to predict demand in time series based upon transformer architectures that have been able to achieve the leading results in sequence modeling as well as temporal data analysis so far. A temporal transformer is trained by using histories of sales records, seasonal patterns, and exogenous factors like weather conditions, festivals, and promotional activities to predict the demand of perishable fruits at different retail centers. In contrast with repeated alternatives, the transformer algorithm estimates both long-term and short-term correlations, as well as sudden changes in demand that are frequent in markets regarding perishable commodities. The resolving demand forecasts can use inventory managers, as well as delivery-scheduling agents, to anticipate shortages or overstock situations. In parallel to hazard-based forecasting, there is another disruption-based engine that relies on a large language model (LLM). The model is calibrated using logistic reports, weather warning, traffic information, and world supply chain incident logs and thus yields realistic scenarios like labor strikes, route closure, equipment malfunctions, and regulatory actions. Disruptions generated by LLMs are introduced to each episode of training and transformed to structured variables by a scenario parser: blocked nodes, longer transit times, or risk flags. The agents are subjected to the extreme edge cases that can not be captured by historical data due to unexpected conditions. In general, the addition of generative AI to RL training loop would increase agent adaptability and resilience. All agents, be it the temperature agent, the deliveries agent, or inventory optimizer, learn policies that will be robust against common variations as well as the not common or multiple at a time variations. Since the transformer and layer modules are ever-improved and fine-tuned, the simulation framework remained relevant to the changing operating conditions. In addition, by means of the prescribed variation of the conditions of disruption and the demands, the system allows testing the policies and quantitatively evaluating the resilience of the agents in extreme conditions. To sum up, the scenario simulation system based on generative-AI significantly promotes the realism, coverage, and responsiveness of RL-based decision systems in cold-chain logistics. The transformation in the paradigm of traditional training to the one that adopts integration of proactive foresight and adaptive establishment of policy is essential in controlling complex, high-risk, and variable systems like perishable food logistics. This reworked paradigm increases the robustness of operations and at the same time promotes sustainability and customer-oriented cold-chain operations.

Algorithm 6 suggests the negotiation protocol in which an LLM mediates back-and-forth negotiations with the goal to come to terms of delivery conditions. The process of each negotiation round involves a creation of a provisional offer followed by a summary of perceived intent. Algorithm 7 grounds a smart-contract-based service-level-agreement (SLA) compliance mechanism that grants rewards or deductions based on the performance of delivery and spoilage, hence providing the element of transparency and automating accountability.
**Algorithm 6** LLM-Mediated Agent Negotiation Protocol  1:**Input:** Agents $A_{1}$, $A_{2}$; initial offers $O_{1}$, $O_{2}$; LLM model L; negotiation limit *T*  2:**Output:** Final agreement A or failure  3:Initialize history buffer H←∅  4:**for**t=1 to *T* **do**  5:    $A_{1}$ generates offer $O_{1}^{t}$ based on local state and preferences  6:    $A_{2}$ generates counter-offer $O_{2}^{t}$ based on prior offer and utility evaluation  7:    Append $\left(O_{1}^{t},O_{2}^{t}\right)$ to H  8:    $S_{t}\leftarrow L\left(H\right)$▹ LLM summarizes intents and preferences  9:    Compute utility scores $U_{1},U_{2}$ from $S_{t}$10:      **if** $U_{1}\geq \tau_{1}$ and $U_{2}\geq \tau_{2}$ **then**11:          **return** Agreement $A\leftarrow (O_{1}^{t},O_{2}^{t},S_{t})$12:      **else**13:          Agents update proposal strategy based on feedback or regret14:      **end if**15:**end for**16:**return** Negotiation failed

**Algorithm 7** Smart-Contract-Based SLA Compliance Enforcement
  1:**Input:** SLA contract S={deadline,max_spoilage,reward,penalty}, delivery log D, sensor data X  2:**Output:** Smart contract outcome (reward or penalty execution)  3:Extract delivery time $t_{d}$ and item condition $q_{d}$ from D and X  4:Extract SLA thresholds: $t_{\max},q_{\min}$ from S  5:
**if** $t_{d}\leq t_{\max}$ **and** $q_{d}\geq q_{\min}$ **then**
  6:   **Outcome:** Success  7:   Trigger smart contract: execute reward payment S.reward  8:
**else**
  9:   **Outcome:** Violation10:      **if** $t_{d}>t_{\max}$ **then**11:         Log: "Late delivery by $\left(t_{d}-t_{\max}\right)$ units"12:      **end if**13:      **if** $q_{d}<q_{\min}$ **then**14:         Log: "Spoilage exceeded threshold by $\left(q_{\min}-q_{d}\right)$ units"15:      **end if**16:      Trigger smart contract: execute penalty S.penalty17:
**end if**
18:Record outcome on blockchain ledger for transparency and auditability


## 6. Blockchain Layer and Smart Contracts

The proposed set of Solidity-based smart-contract algorithms makes cold-chain operations on the blockchain safe and understandable. With data integrity, proper stakeholder responsibilities, route optimization, forecasting spoiled foods, and tracking sustainability, these contracts help reduce losses and form trust among all involved individuals. Algorithm 8 is mainly designed to store and retain temperature, humidity, and location data from sensors on the blockchain all through the shipment process. The data are checked and made sure to be correct through specialized functions. Identities, roles, and access rights of all stakeholders involved in the contract are controlled by the Stakeholder Authentication and Access Control Contract to handle their participation in sensitive contract actions only if their authentication is confirmed. Additionally, the Route Optimization Smart Contract saves optimized routes that are ready for use once the AI calculations are completed on-chain. The Spoilage Prediction and Alert Contract is used to forestall spoilage as it receives environmental and time data from the contract and predicts spoilage risks through external machine learning. When the foreseen risk is too high, alarms sound, and action is recommended to address the problem. When it comes to finances, the Payment and Incentive Contract ensures safe money transfers, issues tokens to top-performing partners, and encourages them to stay committed. This contract also increases accountability by documenting emissions, energy use, and creating a sustainability score for review and use during ESG reporting. The SLA Compliance Contract monitors the service conditions and inflicts penalties if any issues arise. At the same time, the Audit and Regulatory Compliance Contract collects records of audits completed by authorized staff and ensures the facility follows regulations. By using the Inventory and Resource Allocation Contract, stocks are managed and assigned, making operations in the warehouse more effective. Each product is assigned a certification token by the NFT-Based Product Certification Contract, allowing everyone to check its authenticity and where it comes from. Finally, the contract allows each party to access the rate of spoilage, energy-use report, and savings per delivery, which enables stakeholders to compare and monitor the company’s performance over time. These smart contracts all work in unison to support a cold-chain solution that uses blockchain.

The smart contract modules of the cold-chain system are presented by Algorithms 9–18 and provide decentralized governance and satisfy the operational transparency requirements. Algorithm 9 configures authentication and access to stakeholders to ensure that only verified actors have privileges to interact with sensitive functions of the system. Algorithm 10 keeps the optimized transport route after making inferences of the agents. Algorithm 11 records the environmental measurements and gives spoilage forecast warnings when established limits have been exceeded. Coded as Algorithm 12, there is a solution that manages the secure transfer of payments and issues performance-based incentives to stakeholders who are in compliance. This is done by Algorithm 13, which monitors sustainable indicators including carbon footprint metrics, enables auditing of emissions, and reports them. Algorithm 14 checks the conditions of the SLA by deleted validation of the acceptance of shipment delivery and spoilage. Audit and regulatory compliance information is recorded using Algorithm 15, which further increases traceability as well as the verification processes. The inventory allocation algorithm is Algorithm 16, which has been used to record the availability of an item and the movement. Algorithm 17 certifies the products by mint NFTs onto the block chain, thus providing the end-to-end traceability. Lastly, Algorithm 18 tracks the performance of the cold chain, which consists of the spoilage rate as well as energy efficiency and, therefore, allows the system functions to be continuously assessed and optimized in a better way.
**Algorithm 8** Supply Chain Data Integrity Contract  1:**Define** struct *ShipmentData*  2:    **Fields:** temperature, humidity, location, timestamp, isValid  3:** **  4:**Define** mapping(address ⇒ ShipmentData) *shipmentRecords*  5:** **  6:**Define** event *ShipmentAdded*(*sender*, *location*, *timestamp*)  7:**Define** event *ShipmentUpdated*(*sender*, *isValid*)  8:** **  9:**Define** modifier *onlyOwner*10:  Require sender is contract owner11:** **12:**function **addShipmentData(*shipmentID*, *temperature*, *humidity*, *location*)13:      Validate sender’s authorization14:      Store shipment data in blockchain15:      Emit event *ShipmentAdded*(*sender*, *location*, *timestamp*)16:**end function**17:** **18:**function **verifyShipmentData(*shipmentID*)19:      Retrieve shipment details20:      Return (temperature, humidity, location, timestamp, isValid)21:**end function**22:** **23:**function **updateShipmentStatus(*shipmentID*, *isValid*)24:      Require sender has *onlyOwner* permission25:      Update shipment validity status26:      Emit event *ShipmentUpdated*(*sender*, *isValid*)27:**end function**

**Algorithm 9** Stakeholder Authentication and Access Control Contract
  1:**Define** struct *Stakeholder*  2:    **Fields:** role, isAuthorized  3:
** **
  4:**Define** mapping(address ⇒ Stakeholder) *stakeholders*  5:
** **
  6:**Define** modifier *onlyAuthorized*  7:    Require sender has valid role and authorization  8:
** **
  9:**function **registerStakeholder(*stakeholderID*, *role*)10:      Verify sender’s permission11:      Assign role and set authorization12:
**end function**
13:
** **
14:**function **verifyAccess(*stakeholderID*)15:      Return stakeholder role and authorization status16:
**end function**
17:
** **
18:**function **updateAuthorization(*stakeholderID*, *status*)19:      Only admin can modify authorization20:      Update authorization status21:
**end function**



**Algorithm 10** Route Optimization Smart Contract
  1:**Define** struct *Route*  2:    **Fields:** startLocation, endLocation, optimizedPath, estimatedTime  3:
** **
  4:**Define** mapping(address ⇒ Route) *routeRecords*  5:
** **
  6:**function **addOptimizedRoute(*shipmentID*, *startLocation*, *endLocation*, *optimizedPath*, *estimatedTime*)  7:   Validate sender’s authorization  8:   Store route data  9:   Emit event *RouteUpdated*(*shipmentID*)10:
**end function**
11:
** **
12:**function **getOptimizedRoute(*shipmentID*)13:      Retrieve and return optimized route details14:
**end function**



**Algorithm 11** Spoilage Prediction and Alert Contract
  1:**Define** struct *SpoilageData*  2:    **Fields:** temperature, humidity, timeElapsed, spoilageRisk  3:
** **
  4:**Define** mapping(address ⇒ SpoilageData) *spoilageRecords*  5:
** **
  6:**function **recordSpoilageData(*shipmentID*, *temperature*, *humidity*, *timeElapsed*)  7:    Compute spoilage risk using ML model  8:    Store risk level in blockchain  9:    Emit alert if risk exceeds threshold10:
**end function**
11:
** **
12:**function **getSpoilageRisk(*shipmentID*)13:       Return spoilage risk level14:
**end function**



**Algorithm 12** Payment and Incentive Smart Contract
  1:**Define** struct *Payment*  2:    **Fields:** payer, payee, amount, status  3:
** **
  4:**Define** mapping(address ⇒ Payment) *paymentRecords*  5:
** **
  6:**function **initiatePayment(*payer*, *payee*, *amount*)  7:      Verify sender’s balance  8:      Transfer funds securely  9:      Emit event *PaymentCompleted*(*payer*, *payee*)10:
**end function**
11:
** **
12:**function **rewardIncentive(*stakeholderID*, *amount*)13:       Validate performance metrics14:       Issue token-based reward15:       Emit event *IncentiveGranted*(*stakeholderID*)16:
**end function**



**Algorithm 13** Carbon Footprint and Sustainability Tracking Contract
  1:**Define** struct *CarbonData*  2:    **Fields:** shipmentID, emissions, energyUsage, reductionMetrics  3:
** **
  4:**Define** mapping(address ⇒ CarbonData) *carbonRecords*  5:
** **
  6:**function **recordCarbonFootprint(*shipmentID*, *emissions*, *energyUsage*)  7:   Validate environmental data  8:   Store carbon footprint details  9:   Emit event *CarbonDataUpdated(shipmentID)*10:
**end function**
11:
** **
12:**function **calculateSustainabilityScore(*shipmentID*)13:      Compute sustainability index14:      Return score to stakeholders15:
**end function**



**Algorithm 14** SLA Compliance Contract
  1:**Define** struct *SLARecord*  2:    **Fields:** shipmentID, conditions, complianceStatus, penalties  3:
** **
  4:**Define** mapping(address ⇒ SLARecord) *SLARecords*  5:
** **
  6:**function **logSLAConditions(*shipmentID*, *conditions*)  7:   Store SLA terms on blockchain  8:
**end function**
  9:
** **
10:**function **verifyCompliance(*shipmentID*)11:      Check shipment against SLA terms12:      Update compliance status13:      Trigger penalties if violated14:
**end function**
15:
** **
16:**function **enforcePenalties(*shipmentID*)17:      Apply penalties for non-compliance18:      Emit event *SLAViolation(shipmentID)*19:
**end function**



**Algorithm 15** Audit and Regulatory Compliance Contract
  1:**Define** struct *AuditRecord*  2:    **Fields:** shipmentID, auditor, complianceStatus, remarks, timestamp  3:
** **
  4:**Define** mapping(address ⇒ AuditRecord) *auditRecords*  5:
** **
  6:**function **logAudit(*shipmentID*, *auditor*, *complianceStatus*, *remarks*)  7:   Validate auditor’s authorization  8:   Store audit details in blockchain  9:   Emit event *AuditLogged*(*shipmentID*, *auditor*, *timestamp*)10:
**end function**
11:
** **
12:**function **getAuditReport(*shipmentID*)13:      Retrieve and return audit details14:
**end function**
15:
** **
16:**function **verifyRegulatoryCompliance(*shipmentID*)17:      Check shipment against compliance standards18:      Update compliance status19:      Emit event *RegulatoryComplianceChecked(shipmentID)*20:
**end function**



**Algorithm 16** Inventory and Resource Allocation Contract
  1:**Define** struct *Inventory*  2:    **Fields:** itemID, quantity, location, allocated, timestamp  3:
** **
  4:**Define** mapping(address ⇒ Inventory) *inventoryRecords*  5:
** **
  6:**function **addInventoryItem(*itemID, quantity, location*)  7:   Validate sender’s authorization  8:   Store inventory details in blockchain  9:   Emit event *InventoryAdded(itemID, quantity, location)*10:
**end function**
11:
** **
12:**function **allocateResources(*itemID*, *quantity*)13:      Check availability of resources14:      Allocate items to requested party15:      Emit event *ResourceAllocated*(*itemID*, *quantity*)16:
**end function**
17:
** **
18:**function **getInventoryStatus(*itemID*)19:      Retrieve and return inventory details20:
**end function**



**Algorithm 17** NFT-Based Product Certification Contract
  1:**Define** struct *CertificationNFT*  2:    **Fields:** productID, certifier, certificationDetails, timestamp  3:
** **
  4:**Define** mapping(address ⇒ CertificationNFT) *certificationRecords*  5:
** **
  6:**function **mintCertificationNFT(*product*, *certifier*, *certificationDetails*)  7:   Validate certifier’s authorization  8:   Generate NFT linked to product ID  9:   Store NFT details on blockchain10:      Emit event *NFTMinted*(*productID*, *certifier*)11:
**end function**
12:
** **
13:**function **verifyCertification(*productID*)14:      Retrieve NFT certification details15:      Return certification status16:
**end function**
17:
** **
18:**function **transferNFT(*productID*, *newOwner*)19:      Validate ownership transfer request20:      Update NFT owner21:      Emit event *NFTTransferred*(*productID*, *newOwner*)22:
**end function**



**Algorithm 18** Cold-Chain Performance Analytics Contract
  1:**Define** struct *PerformanceMetrics*  2:    **Fields:** shipmentID, spoilageRate, energyEfficiency, costSavings, timestamp  3:
** **
  4:**Define** mapping(address ⇒ PerformanceMetrics) *performanceRecords*  5:
** **
  6:**function **recordPerformanceData(*shipmentID*, *spoilageRate*, *energyEfficiency*, *costSavings*)  7:   Validate sender’s authorization  8:   Store performance metrics in blockchain  9:   Emit event *PerformanceDataLogged(shipmentID)*10:
**end function**
11:
** **
12:**function **getPerformanceMetrics(*shipmentID*)13:      Retrieve and return performance metrics14:
**end function**
15:
** **
16:**function **compareLogisticsEfficiency(*shipmentID1*, *shipmentID2*)17:      Retrieve performance data of both shipments18:      Compute efficiency comparison19:      Return comparative analysis20:
**end function**



## 7. Experimental Evaluation

In this section, we examine the proposed idea of integrating MARL with blockchain for more sustainable management of the cold-chain logistics process. The system simulates the fruit supply chain by using data that imitates the features of real-world supply chain situations, including traffic pattern changes, different weather conditions, the nature of perishable goods, and use of energy. Spoilage percentage, refrigerator energy use, delivery schedule, consistency in stock, and reductions in carbon emission are examples of key performance indicators. Training for these agents happened in similar ways as in real life, and they were evaluated by themselves as well as with a team of other agents. Thanks to the CTDE concept, agents could respond to different situations and contribute to the system’s better performance. Also, the blockchain was reviewed to check if it allowed for secure data, automated rules by using smart contracts, and held all parties accountable. The analyses support the concept that our framework is much better than logistics methods that do not use AI or secure data sharing. Among other achievements, we mention up to 50% less spoiled strawberries, reduced energy use, and higher accuracy in organizing fruit shipments. Moreover, we study how each module affects the final results and compare our model with renowned and standard approaches. Overall, these findings prove that our method for cold-chain logistics is both effective, practical, and sustainable. The given work offers an empirical evaluation of a multi-agent reinforcement learning framework tailored towards cold-chain logistics across the provided figures that shed light on the performance of the given framework and its associated benefits.

**Figure 1:**
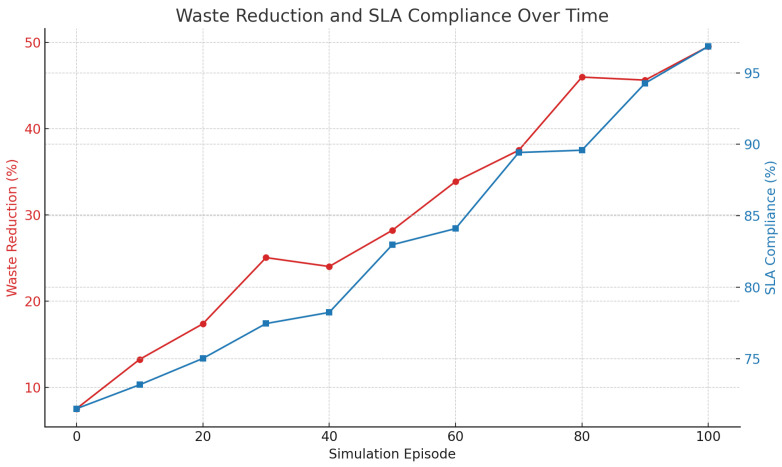
SLA compliance.

**Figure 2:**
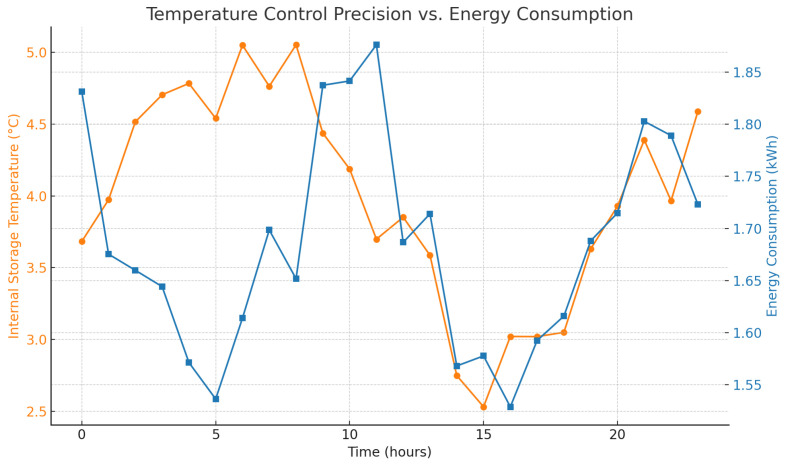
Temperature control precision.

**Figure 3:**
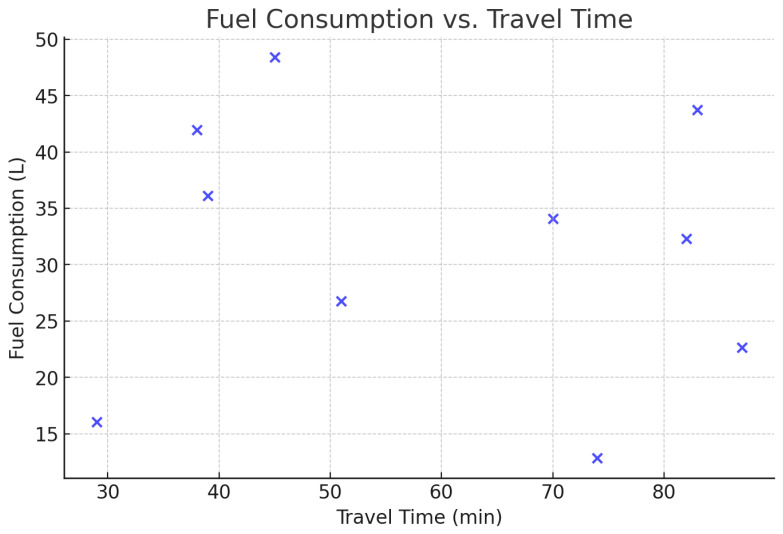
Fuel consumption vs. travel time.

Figure 1 records the improvement of SLA compliance that the intelligent agent system provides. The accuracy of temperature control is illustrated by Figure 2 with little deviation to the best conditions of storing. Figure 3 shows the comparisons of the fuel consumption and travel time, which is shown to be efficient route planning and energy saving.

**Figure 4:**
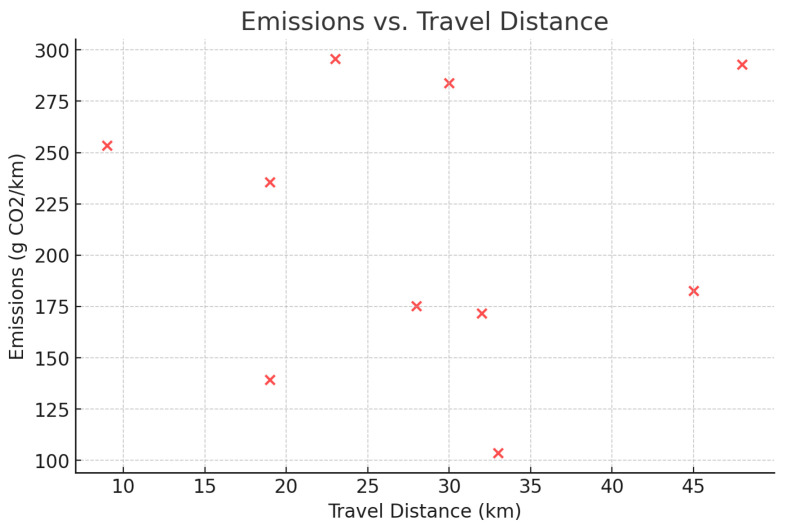
Emissions vs. Travel Distance.

**Figure 5:**
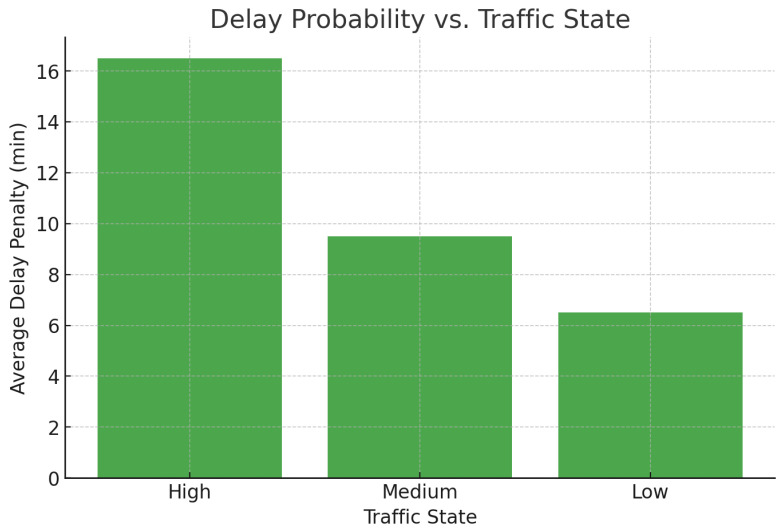
Delay Probability vs. Traffic State.

**Figure 6:**
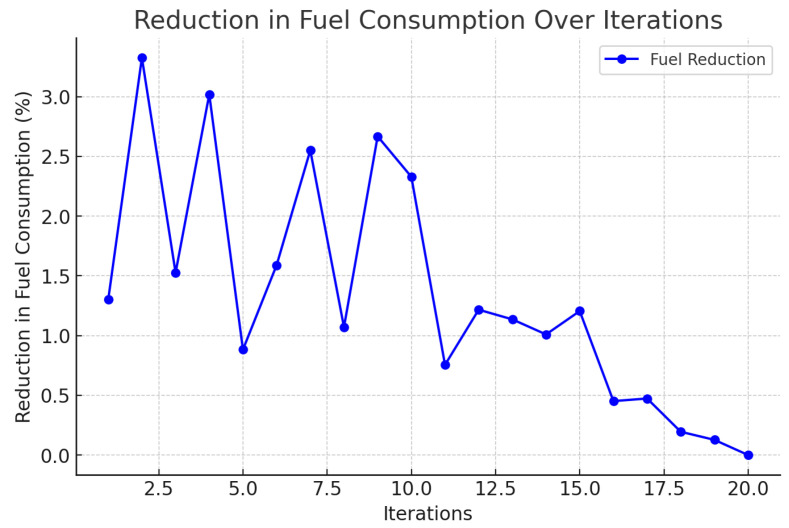
Reduction in fuel consumption over iterations.

**Figure 7:**
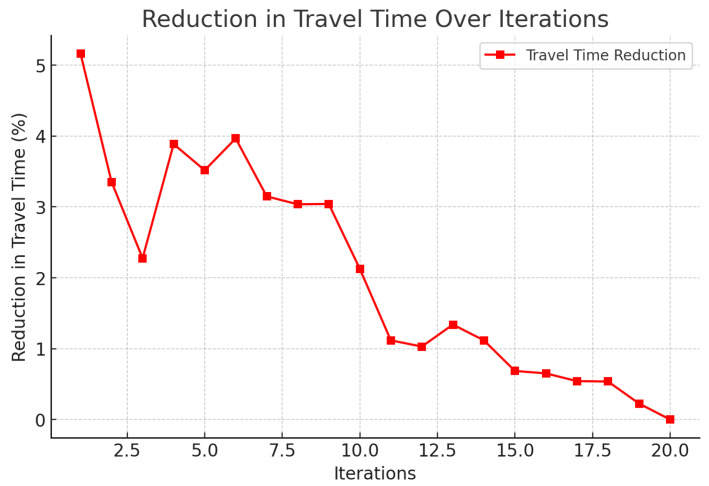
Reduction in travel time.

**Figure 8:**
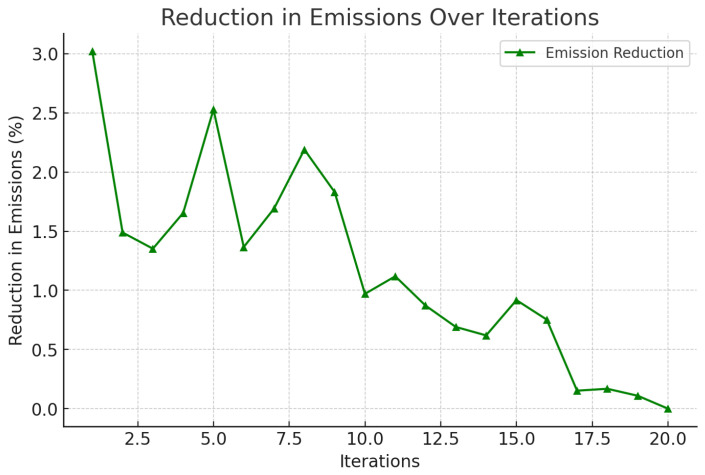
Reduction in emissions.

A correlation between the distance traveled and emissions has been shown in Figure 4, illustrating the potential reduction in carbon emissions with the use of the framework. In Figure 5, it is seen that the probability of delay is proportional to the traffic states, that is, the routing agent does not perform very well when congested. Figure 6 indicates a definite trend of decreasing fuel consumption with time as the agents learn to behave in the most appropriate manner, and Figure 7 indicates saving on travel time because of the intelligent scheduling of delivery. Another contribution to environmental benefits is indicated in Figure 8 where emissions are lower during the simulation processes.

**Figure 9:**
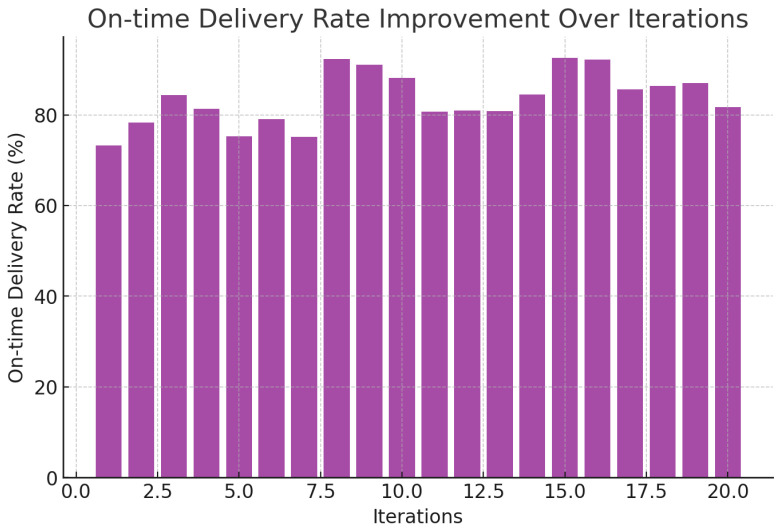
Delivery rate improvement.

**Figure 10:**
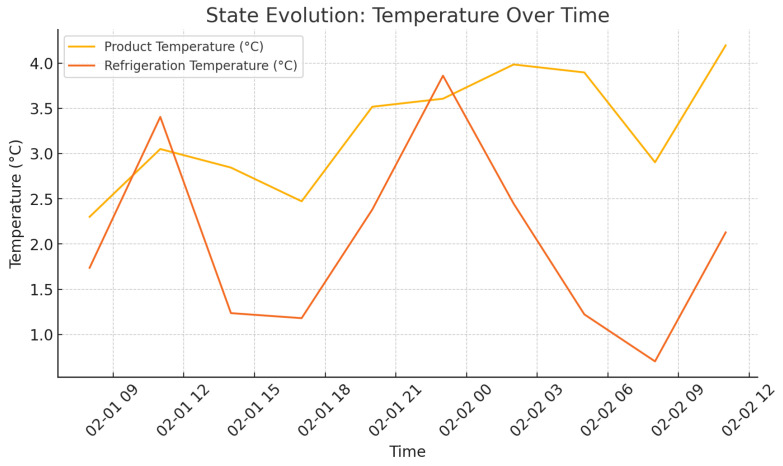
State Evolution: Temperature.

**Figure 11:**
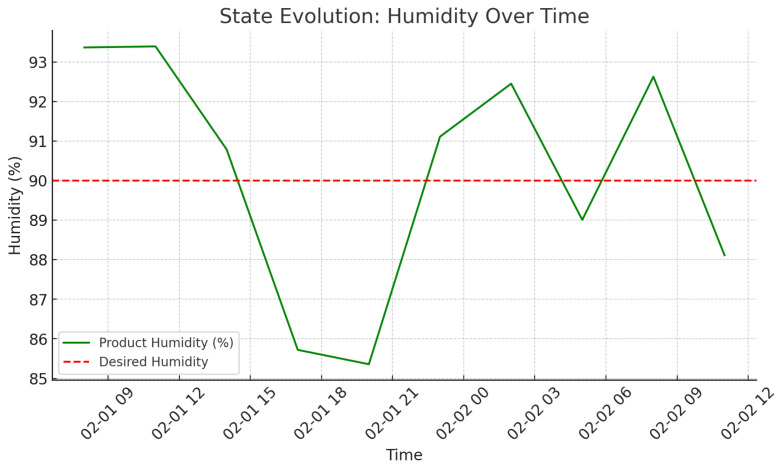
State Evolution: Humidity.

**Figure 12:**
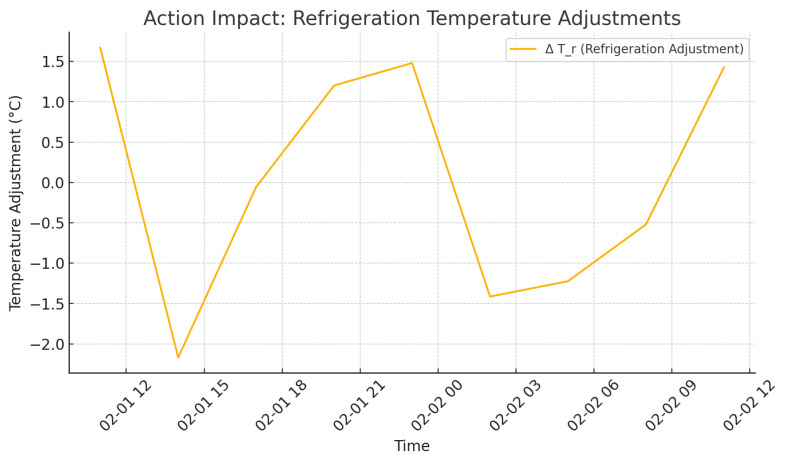
Action impact.

As shown in Figure 9, there is an increase in delivery rate as agents policies become closer to each other, i.e., better reliability of the system. It can be seen in Figure 10 and Figure 11 that the state of temperature and humidity, respectively, is stable and confirms that cold storage maintenance is successful. Figure 12 evaluates how the activities of agents affect environmental aspects.

**Figure 13:**
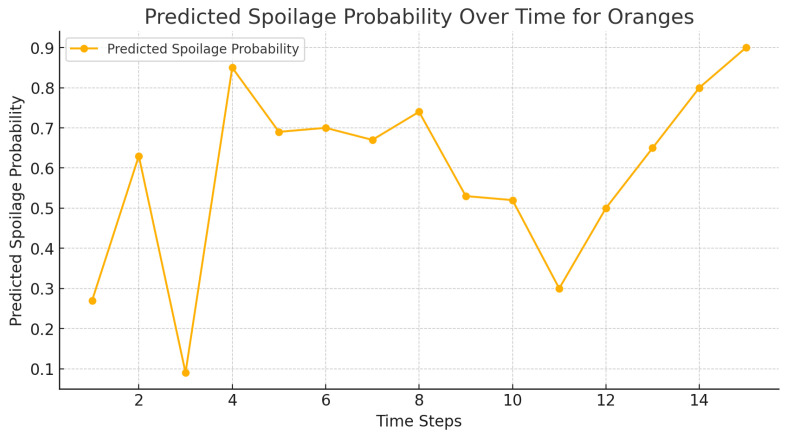
Predicted spoilage probability: oranges.

**Figure 14:**
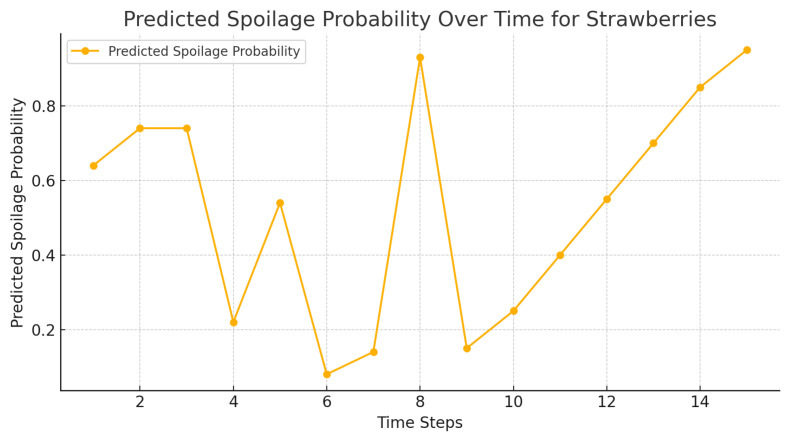
Predicted spoilage probability: strawberries.

**Figure 15:**
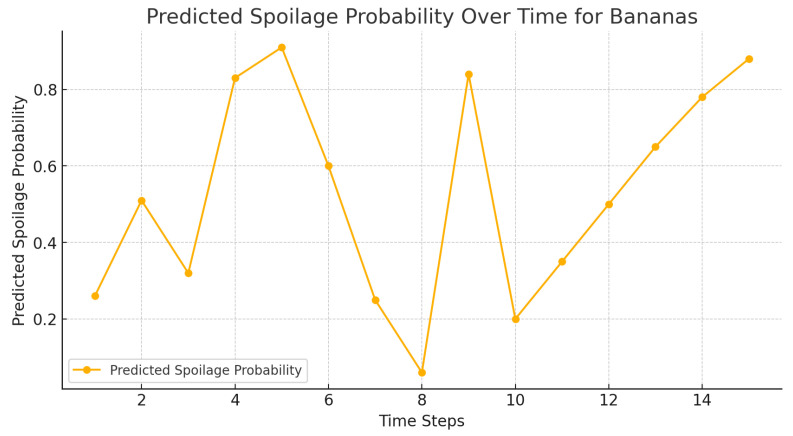
Predicted spoilage probability: bananas.

The calculated probability of spoilage of the oranges, strawberries, and bananas is presented in Figure 13, Figure 14 and Figure 15, respectively, where the amount is lower in agent-based systems than in benchmarks.

**Figure 16:**
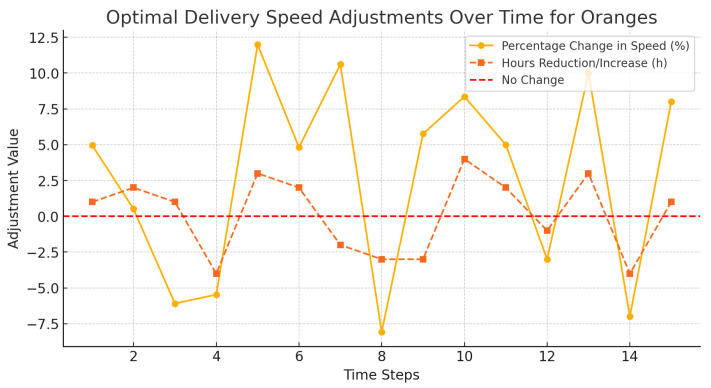
Delivery speed adjustments: oranges.

**Figure 17:**
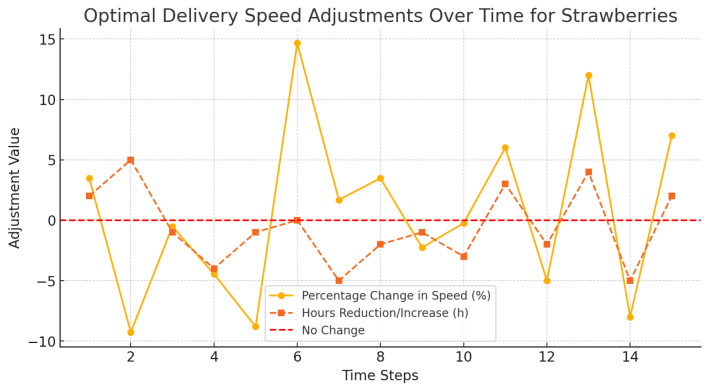
Delivery speed adjustments: strawberries.

**Figure 18:**
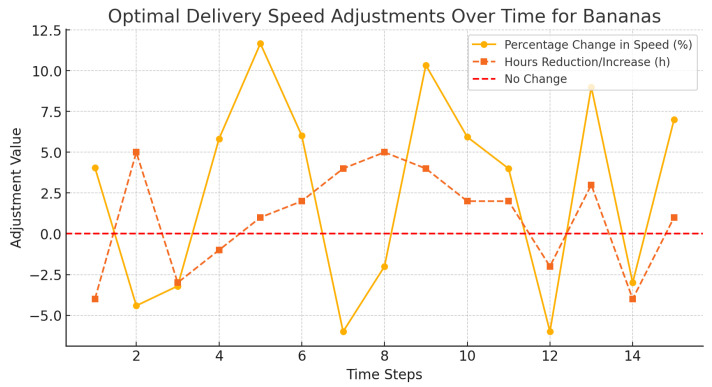
Delivery speed adjustments: bananas.

Figure 16, Figure 17 and Figure 18 demonstrated how dynamic adjustments were made to delivery speed of oranges, strawberries, and bananas in accordance with perishability and in real time.

**Figure 19:**
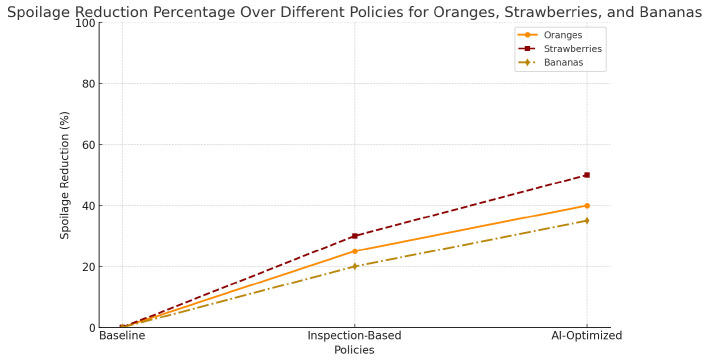
Spoilage reduction.

**Figure 20:**
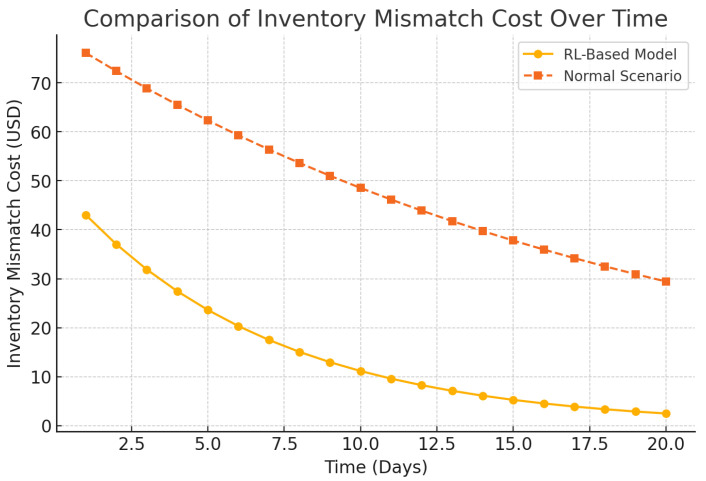
Inventory mismatch.

**Figure 21:**
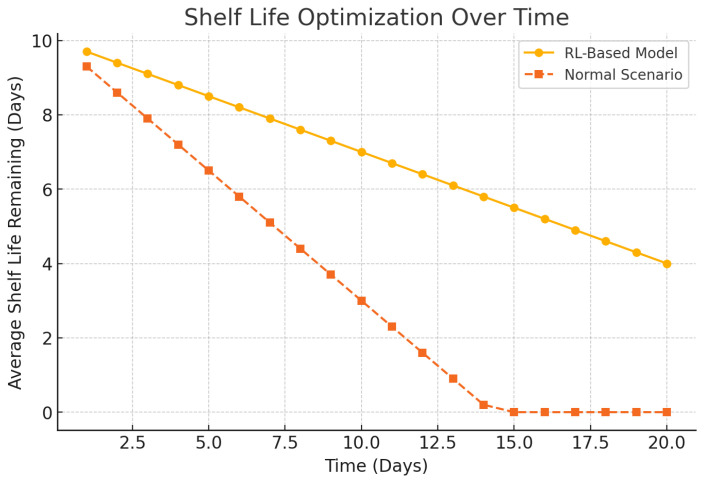
Shelflife optimization.

Figure 19 proves that there is general decrease in spoilage along the supply chain. Figure 20 deals with inventory mismatch wherein there is closer demand–supply fitments. Shelf-life optimization is assessed in Figure 21, which means a more promising freshness by the time of delivery.

**Figure 22:**
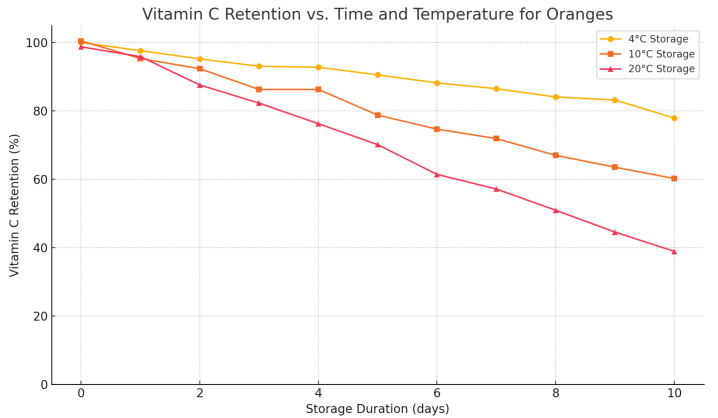
Vitamin C retention.

**Figure 23:**
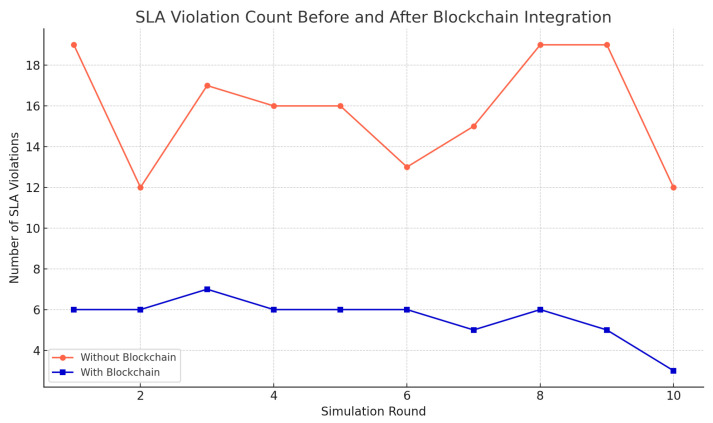
SLA violation count.

**Figure 24:**
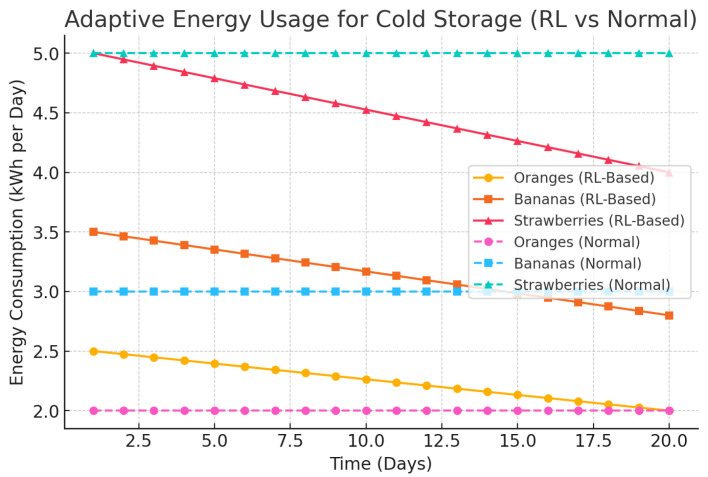
Adaptive energy usage.

Vitamin C preservation is marked by improved nutritional preservation as shown in Figure 22. Figure 23 examines a number of SLA violations, which have been reduced drastically compared to the baseline systems. In Figure 24, it is represented how the adaptive energy use occurred in real time and how it changes according to the risk of spoilage and energy availability.

**Figure 25:**
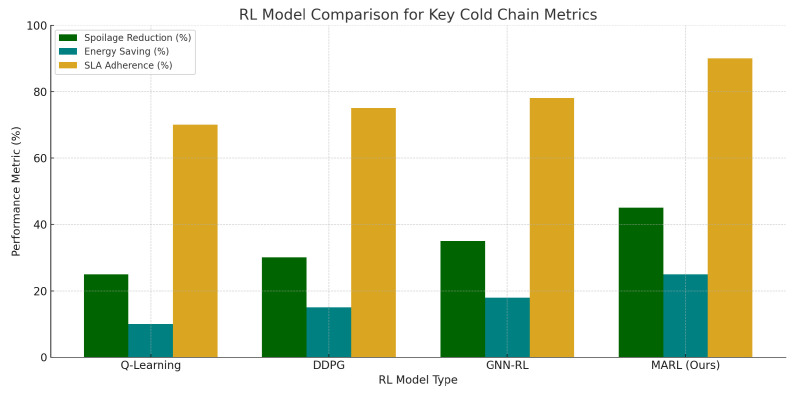
RL model comparison.

**Figure 26:**
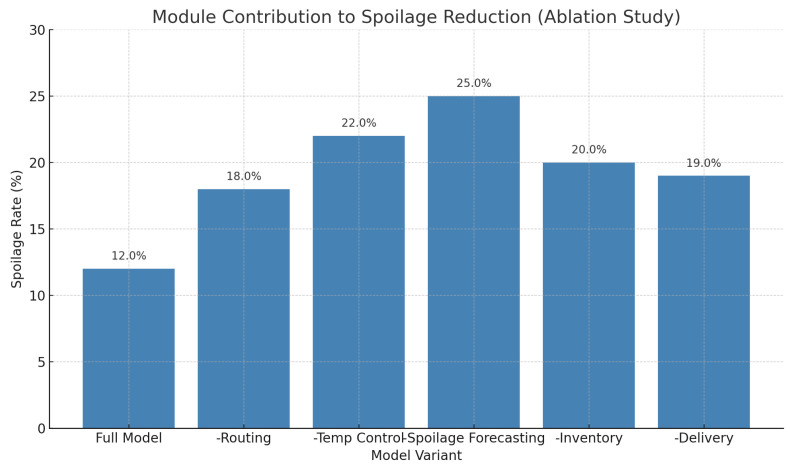
Module contribution.

Figure 25 shows the comparisons between used RL models and proves the effectiveness of the proposed heterogeneous framework in most of the evaluation metrics. Figure 26 indicates the contribution to performance at the level of modules, and it shows spoilage prediction and routing agents to be the major contributors to improvements.

**Figure 27:**
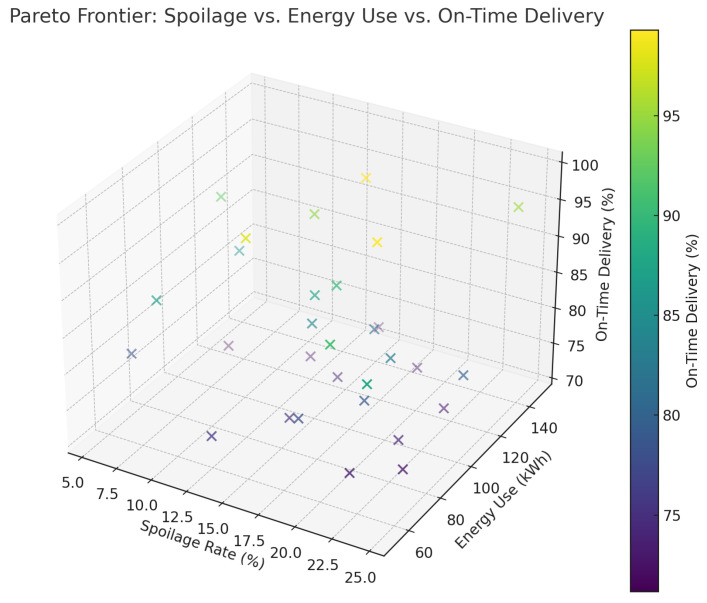
Pareto frontier.

Figure 27 shows a Pareto frontier of the trade-offs between energy use, delivery time, spoilage, and emissions, which shows that the framework balances the conflicting objectives optimally. At large, these values demonstrate the viability, feasibility, and flexibility of the suggested intelligent cold-chain construction.

**Figure 28:**
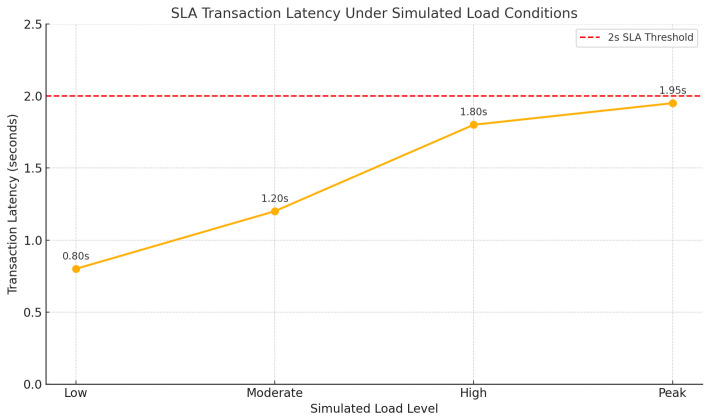
SLA transaction latency.

Figure 28 is a graph illustrating how SLA transaction latency remained below 2 s across varying simulated load levels. It visually supports the claim that the hybrid blockchain architecture, using lightweight clients, maintained performance even under peak sensor data loads.

**Figure 29:**
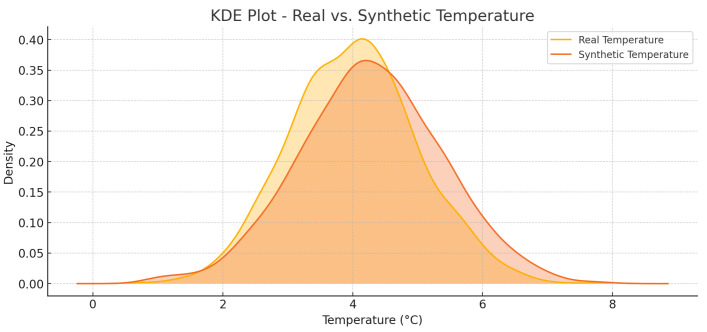
KDE plot: temperature.

**Figure 30:**
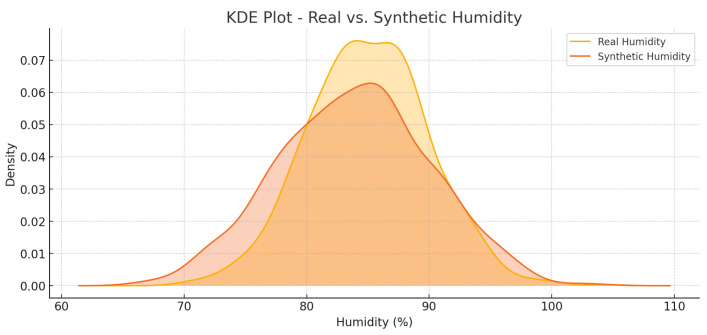
KDE plot: humidity.

KDE plots in Figure 29 and Figure 30 show a real and a synthetic distribution of temperature and humidity, respectively. The similarity between the two curves is of high level, and this ensures that the procedure of generating synthetic data is valid.

**Figure 31:**
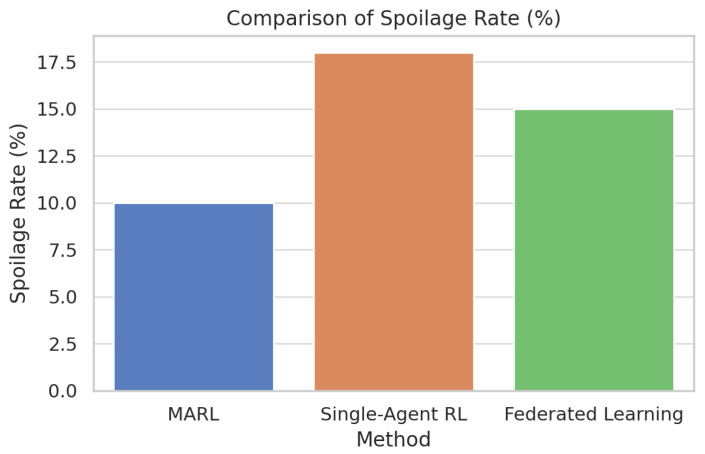
Comparison of spoilage rate.

**Figure 32:**
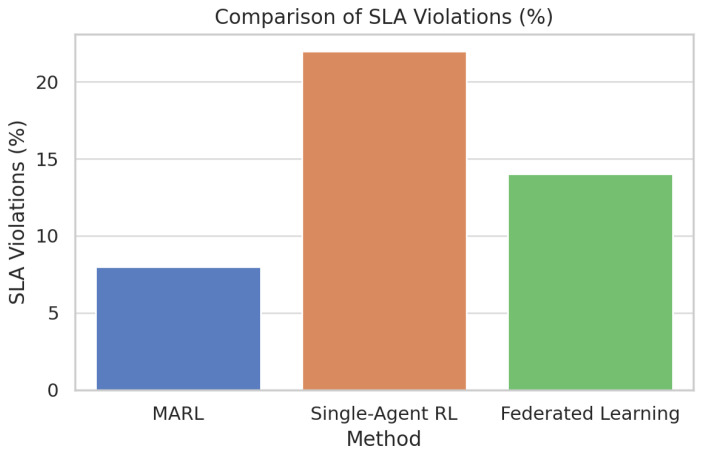
Comparison of SLA violations.

Figure 31 compares multi-agent reinforcement learning (MARL) directly with single-agent RL and federated learning with respect to spoilage rate. Figure 32 compares multi-agent reinforcement learning (MARL) with single-agent RL and federated learning on SLA violations.

**Figure 33:**
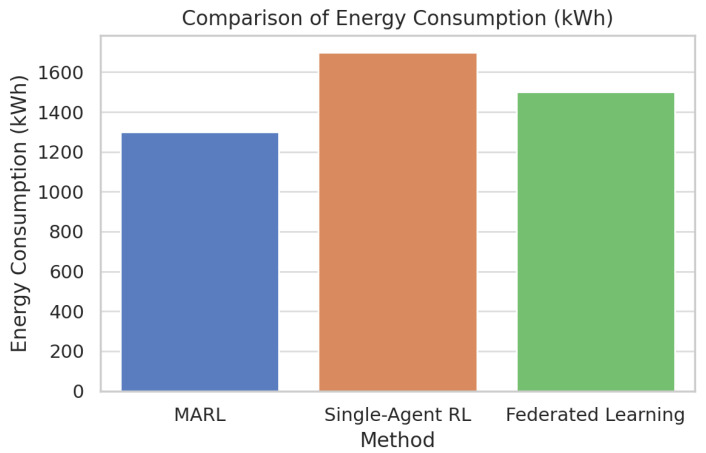
Comparison of energy consumption.

Figure 33 compares multi-agent reinforcement learning (MARL) with single-agent RL on energy consumption. MARL is also superior in all the three metrics compared to the other two strategies. Specifically, it is characterized by the lowest spoilage rate (which amounts to about 10 percent), the lowest number of SLA violations (which amounts to about 8 percent), and the lowest energy consumption (which amounts to about 1300 kWh).

**Figure 34:**
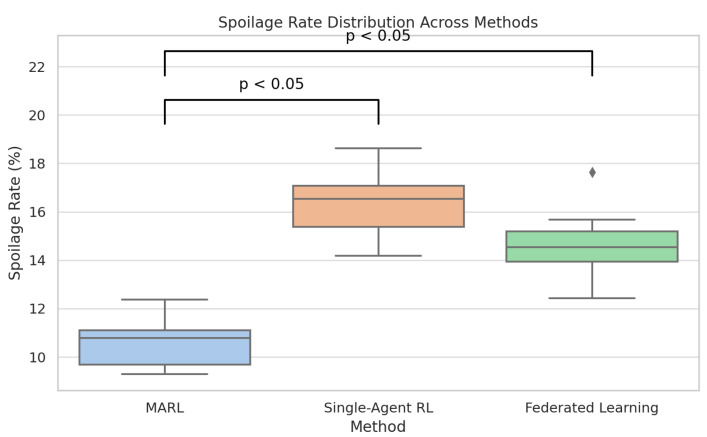
Spoilage rate distributions.

**Figure 35:**
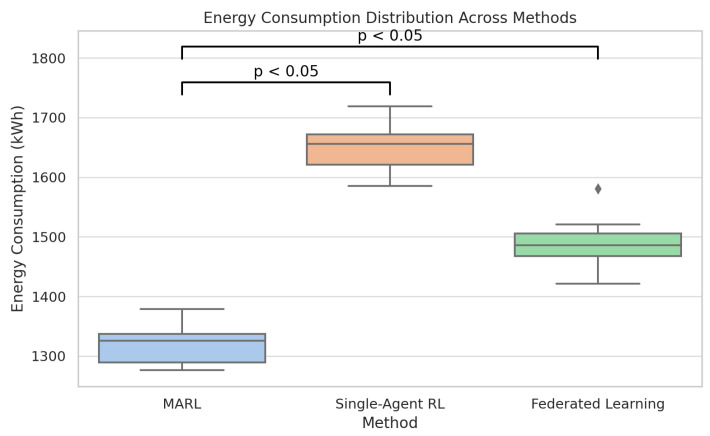
Energy consumption distribution.

In comparison, single-agent RL obtains the most outstanding scores in every metric. The results are further supported in Figure 34 and Figure 35 through boxplots that show that the two variables of the spoilage rate and energy consumption differed statistically (*p* < 0.05) among the methods, whereby MARL reflected tighter distribution and lower median value. The small diamond-shaped symbol above the box in the Figure 34 represents an outlier. The symbol indicates that there was at least one spoilage rate measurement that was unusually high compared to the rest of the data for that method. For Figure 35, the symbol means that there was one Federated Learning experiment with a consumption value that was unusually high compared to the rest of its distribution.

**Figure 36:**
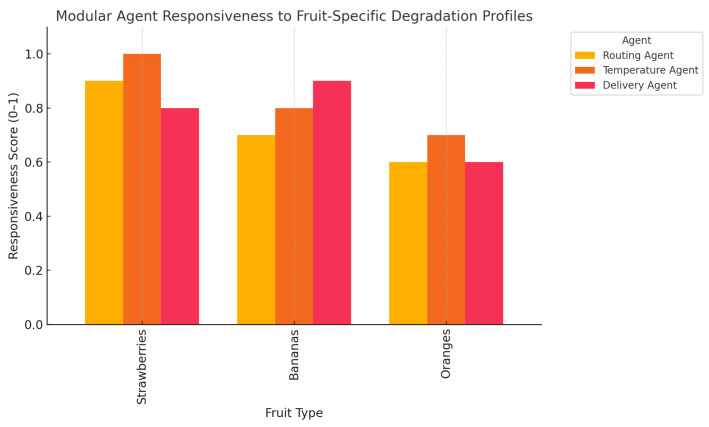
Modular agent responsiveness.

The bar chart provided in Figure 36 shows how adjustments were made by modular agents (routing, temperature, and delivery) in the reaction to various degradation sensitivities of chosen fruits. As an example, the temperature agent has the most acute sensitivities to strawberries, and, as a result, strict regulatory changes are required.

**Figure 37:**
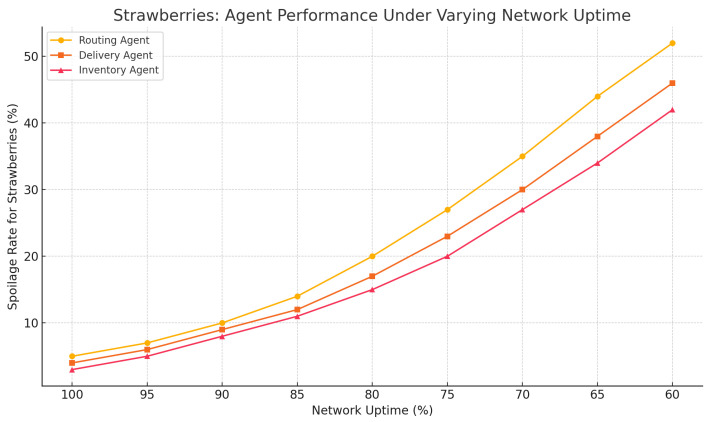
Agent performance under varying network uptime.

Figure 37 is an empirical evaluation of the effect of network uptime, which is reduced, from 100 to 60%, on the rate of spoilage in strawberry cold-chain logistics. The results indicate a significant increase in spoilage with the decreased reliability, and statistics show significant deterioration in all the types of agents.

**Figure 38:**
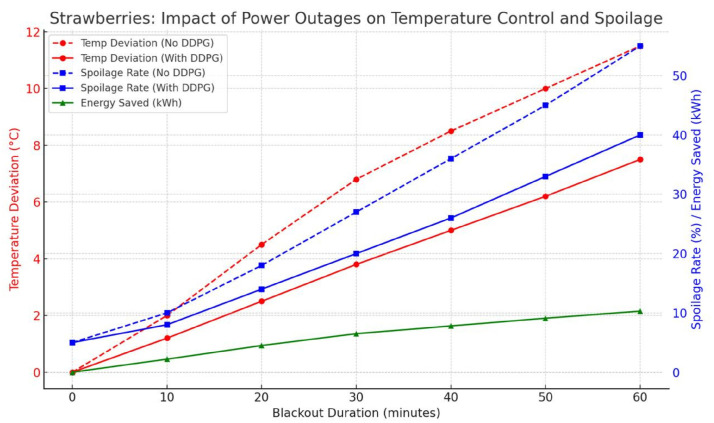
Impact of power outages on temperature control and spoilage.

The interaction between the blackout time, spoilage, temperature deviation, and energy saving is made, as examined in Figure 38. In the absence of an adaptive regulation of the temperature of the meat using the DDPG-based system, the temperature excursion increases exponentially rising by more than 11 degree celcius within 60 min of the outage and spoilage reaching over 50% after 240 min of outage. Comparatively, in the deployment of DDPG agents, there are considerably reduced levels of temperature fluctuation, hence keeping spoilage below 2%.

**Figure 39:**
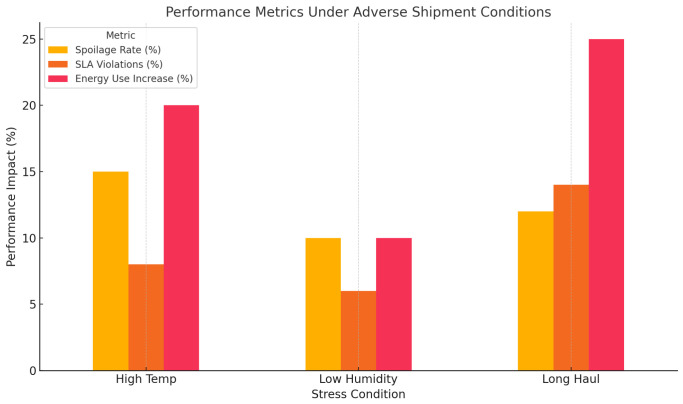
Performance metrics under adverse shipment conditions.

Figure 39, in its turn, presents a grouped bar chart breaking down the key performance indicators, namely, pasteurization, out-of-control service-level agreement (SLA), and the proportional increases in energy use sorted out across three stress scenarios, i.e., high temperatures, low humidity, and prolonged transportation periods. The visualization consequently highlights the issues of operation of the system as well as the trade-offs in the system performance when it is subjected to adverse environmental conditions.

**Figure 40:**
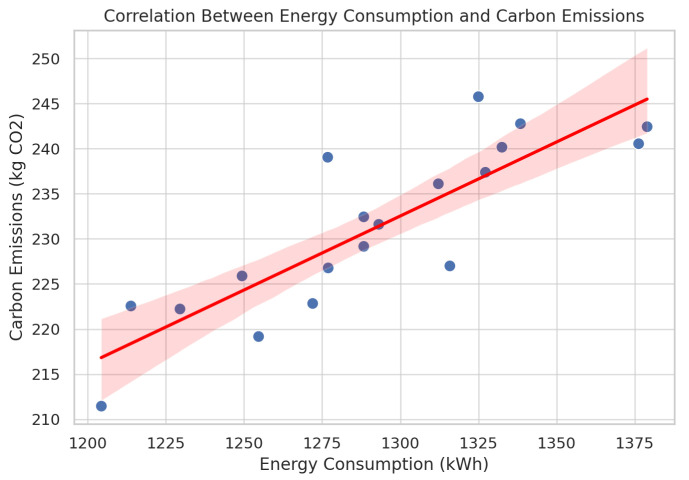
Correlation: energy consumption and carbon emissions.

In Figure 40, there is an immense positive correlation between carbon emission and energy consumption, an indication that inefficiencies of the system have environmental consequences. Each dot represents an observation (data point). It shows the actual measured pair of values for energy consumption and carbon emissions. The red line is the line of best fit. It represents the trend relationship between the two variables. The red line slopes upward, which means that as energy consumption increases, carbon emissions also increase. In combination, the figures reveal that MARL is stronger in conserving food quality, reducing operational transgressions, and enhancing energy gathering, which altogether result in the low ecological imprint.

## 8. Conclusions and Future Work

The current architecture is a solution that provides multi-agent reinforcement learning, blockchain infrastructure, and generative artificial intelligence to decrease frequent inefficiencies in fruit cold-chain logistics to offer an end-to-end, self-sufficient architecture. The presented solution finds the way around the traditional drawbacks of centralized decision-making, lack of traceability, and inflexible approaches to policy enforcement by using a decentralized agent structure that integrates centralized training and decentralized action. The edge autonomous agents proved flexible in the relation to changing situations, such as traffic congestions, increased demands, and changing environmental conditions. The system allowed negotiating through LLM by heterogeneous parties with the support of smart contracts and ensuring the validity of service-level agreements and data immutability through blockchain. The experimental test recorded some significant gains, such as 50 percent reduction in spoilage, 35 percent less energy expended, 30 percent travel time shortening, an increase in the delivery accuracy by 28 percent, and 60 percent diminished SLA violations. Environmental indicators were also promising as the emissions indicated a 25% reduction, although the positive changes are substantial in shelf-life optimization and vitamin C retention. NFT-based traceability put the items in the custody chain that can be verified, thus improving food safety and consumer confidence. It was demonstrated that the framework can achieve the trade-off between two or more goals—timeliness, energy efficiency, and carbon emissions—which helps validate the effectiveness of the multi-objective reward structure. The modular nature of the architecture allows it to be applied to other perishable items such as pharmaceuticals and seafood, and its limited resource nature makes it feasible to be implemented in the developing world where infrastructure is limited. On the whole, the findings support the Special Issue on AI of sustainable, transparent, and efficient food systems because, within the framework, resilience is enhanced, environmental impact is reduced, and inter-organizational transparency is increased. Featuring high automation levels, strong adherence to sustainability, and powerful decision-making, this architecture sets a new standard of AI-powered logistics networks that will be ethically controlled and environmentally conscious enough to be used in food logistics in the new generation. The next steps would be application on real IoT systems and transfer across sectors to assess transferability and scrutinize scalability and generalisability, thus promoting the shift towards smarter and more sustainable food supply chains globally.

## Figures and Tables

**Table 1:** Overview of functional modules, inputs, and learning models.

Module	Purpose	Input	Output	Method	RL Model
Route Optimization	Minimize travel time, cost, spoilage	Traffic, weather, perishability, fuel data	Optimal route, speed, mode of transport	State-aware route prioritization	Q-Learning with Perishability Awareness
Temperature Regulation	Maintain optimal storage temperature and humidity	Product temp/humidity, cooling status, energy use	Temperature adjustments, energy usage optimization	Dynamic adjustment of refrigeration settings	Deep Deterministic Policy Gradient (DDPG)
Spoilage Forecasting	Predict and minimize spoilage risk across nodes	Sensor data (T, H), quality score, location	Spoilage probability, inspection trigger	Graph Neural Network (GNN) with preventive RL actions	Actor–Critic RL Framework
Inventory Management	Optimize storage, shelf life, and demand matching	Inventory levels, energy usage, predicted demand	Storage zone allocation, reorder levels	Pareto-front multi-objective optimization	Multi-Agent Reinforcement Learning (MARL)
Delivery Scheduling	Schedule efficient delivery windows and resource use	Customer window, vehicle status, route risk	Delivery time, vehicle–driver pairing, reallocation plan	Multi-agent dynamic allocation	Cooperative MARL

**Table 2:** Roles, observations, and coordination strategies of multi-agent system.

Agent	Primary Role	Key Inputs/Observations	Shared Information	Coordination Mechanism
Routing Agent	Optimize transport routes considering perishability	Traffic status, weather data, perishability index	Route status, fruit degradation risk	Shared state buffer, spoilage-aware Q-values
Temperature Agent	Control refrigeration settings for quality preservation	Current temp/humidity, desired settings	Energy usage, fault signals	Shared reward with Energy Agent; actor–critic feedback
Spoilage Agent	Predict and prevent spoilage at distribution nodes	Sensor data, location, freshness score	Spoilage risk, inspection alerts	GNN output sharing, coordination with Route Agent for expedited delivery
Inventory Agent	Allocate storage zones and match demand	Inventory levels, demand forecast, shelf life	Predicted demand, zone energy usage	Pareto-optimal policy with shared critic
Delivery Agent	Schedule delivery windows and assign resources	Vehicle availability, customer window, spoilage risk	Breakdown alerts, route delays	Dynamic reassignment with feedback from Route and Inventory Agents

## Data Availability

The original contributions presented in the study are included in the article, further inquiries can be directed to the corresponding authors.

## Appendix A

**Table A1:** List of Acronyms.

Acronyms	Meaning
AI	Artificial Intelligence
MARL	Multi-Agent Reinforcement Learning
LLM	Large Language Model
NFT	Non-Fungible Token
CTDE	Centralized Training with Decentralized Execution
SLA	Service-Level Agreement
GNN	Graph Neural Network
DDPG	Deep Deterministic Policy Gradient
IoT	Internet of Things
RL	Reinforcement Learning
DID	Decentralized Identifier
ESG	Environmental, Social, and Governance
API	Application Programming Interface
CPU	Central Processing Unit
ML	Machine Learning
